# Strengthening open disclosure after incidents in maternity care: a realist synthesis of international research evidence

**DOI:** 10.1186/s12913-023-09033-2

**Published:** 2023-03-27

**Authors:** Mary Adams, Julie Hartley, Natalie Sanford, Alexander Edward Heazell, Rick Iedema, Charlotte Bevan, Maria Booker, Maureen Treadwell, Jane Sandall

**Affiliations:** 1 Department of Women and Children’s Health, School of Life Course and Population Sciences, King’s College London, St Thomas’ Hospital, Westminster Bridge Road, London, SE1 7EH UK; 2 The Florence Nightingale Faculty of Nursing, Midwifery, and Palliative Care, King’s College London, London, UK; 3 School of Medical Sciences, University of Manchester, Manchester, UK; 4 School of Life Sciences and Medicine, King’s College London, London, UK; 5 The Stillbirth and Neonatal Death Charity (SANDS), London, UK; 6 BirthRights, London, UK; 7 The Birth Trauma Association, London, UK

**Keywords:** Open disclosure, Adverse events, Incident reviews, Family involvement, Realist evaluation, Realist literature synthesis, Maternity safety, Patient-centred care, Healthcare safety, Medical error

## Abstract

**Background:**

Open Disclosure (OD) is open and timely communication about harmful events arising from health care with those affected. It is an entitlement of service-users and an aspect of their recovery, as well as an important dimension of service safety improvement. Recently, OD in maternity care in the English National Health Service has become a pressing public issue, with policymakers promoting multiple interventions to manage the financial and reputational costs of communication failures. There is limited research to understand how OD works and its effects in different contexts.

**Methods:**

Realist literature screening, data extraction, and retroductive theorisation involving two advisory stakeholder groups. Data relevant to families, clinicians, and services were mapped to theorise the relationships between contexts, mechanisms, and outcomes. From these maps, key aspects for successful OD were identified.

**Results:**

After realist quality appraisal, 38 documents were included in the synthesis (22 academic, 2 training guidance, and 14 policy report). 135 explanatory accounts were identified from the included documents (with *n* = 41 relevant to families; *n* = 37 relevant to staff; and *n* = 37 relevant to services). These were theorised as five key mechanism sets: (a) meaningful acknowledgement of harm, (b) opportunity for family involvement in reviews and investigations, (c) possibilities for families and staff to make sense of what happened, (d) specialist skills and psychological safety of clinicians, and (e) families and staff knowing that improvements are happening. Three key contextual factors were identified: (a) the configuration of the incident (how and when identified and classified as more or less severe); (b) national or state drivers, such as polices, regulations, and schemes, designed to promote OD; and (c) the organisational context within which these these drivers are recieived and negotiated.

**Conclusions:**

This is the first review to theorise how OD works, for whom, in what circumstances, and why. We identify and examine from the secondary data the five key mechanisms for successful OD and the three contextual factors that influence this. The next study stage will use interview and ethnographic data to test, deepen, or overturn our five hypothesised programme theories to explain what is required to strengthen OD in maternity services.

**Supplementary Information:**

The online version contains supplementary material available at 10.1186/s12913-023-09033-2.

## Introduction and background

Open disclosure (OD) is the open and timely communication with a patient or family about an incident that resulted in harm during their care. The principles for conducting OD have remained unchanged for almost 30 years [1–3] and OD has been increasingly recognised as an entitlement of service users, a necessity for many injured patients, and a valuable aspect of organisational improvement internationally [4–7]. Harmed patients’ experiences have been identified as valuable learning resources for professionals and services [8, 9]. For families, OD is expected to offer insight into areas of poor care as well as reduce their felt alienation and anger with a clinician or a service that might have failed them [10].

OD expectations and practices in maternity care surface a series of social, organisational, professional, and personal issues that are more acute than in most other clinical areas. This is in part because maternity care involves complex and episodic care pathways and a service that must respond to rapid and unpredictable demand [11]. The historical organisation of maternity care into ‘high’ and ‘low’ risk systems is challenging when outcomes in maternal care are often unpredictable [12]. The pace and complexity of service delivery can result in notable gaps in care and communication, [12] including gaps post-incident. Second, clinicians can face unique challenges around consent and shared decision-making in maternity care, especially in delivery suite settings, where many unanticipated incidents of harm occur [13]. Furthermore, in a clinical speciality where “the cost of harm can be catastrophic” [13], many families and healthcare staff reflect a widespread social view that modern childbirth is “largely free from complications” [12]. The challenges of initiating disclosure in a service characterised by “high expectations and unpredictability” have been noted previously ([14], p1).

In addition to these distinctive socio-emotional aspects of care delivery, in modern organisations and across many legal systems, incidents in maternity care are notable for their high reputational costs to services, personal and professional costs to staff, and high total financial burden on services [13–19]. For example, in England in 2021–2022, although legal claims for compensation for avoidable injury in maternity care were relatively low (12% by volume of NHS claims), the costs of these claims amounted to over 62% of all secondary care claims because they are connected to the ongoing costs of care for a disabled child [18]. The overall escalating costs of managing and compensating maternity claims in secondary care is now forecast to greatly exceed the amount of money spent on delivering all babies [20], constituting a significant threat to the sustainability of publicly funded health care in England and Wales [21]. The need to manage these costs has generated a series of financially-incentivised measures for health organisations to drive safety improvement and the involvement of injured families in maternity services [17, 18].

In maternity and other clinical areas in some countries, a significant issue affecting OD is the introduction of regulations to drive candour practices within healthcare organisations [22]. In 2014, a statutory Duty of Candour (DoC) was introduced in the National Health Services (NHS) in England and Wales with The Health and Social Care Act of 2008 (Regulated Activities) Regulations 2014, Regulation 20 [23]. An equivalent duty was introduced in Scotland in 2018 [24]. These were to supplement the professional responsibilities of clinicians, to establish organisational accountability around being open with patients following harm in healthcare, and to place the 2009 National Patient Safety Agency guidance on ‘Being Open’ for services on a legal footing [25]. The guidance covers the entire disclosure process, from truthfulness and apology to the provision of professional support, local incident reporting and investigation, and provision of ongoing care. A year after its publication, in England, the Morecambe Bay Investigation Report [26] made a powerful case for a statutory duty of acknowledgement and honesty in maternity services, highlighting the need for families to be informed of serious incidents affecting them and their entitlement to explanation [27]. Since then, NHS maternity services have been the focus of a raft of policy directives to enhance openness, to improve engagement with families, and to learn from preventable deaths and serious injury [28–33]. This focus is driven, in part, by the escalating costs of litigation and claims settlements for serious injury during maternity care [31, 32], as well as by public scandals like Morecambe Bay and the pressure of patient activists for the NHS to improve safety in maternity care. There is some evidence from national reviews that the incidence of OD with families, or at least the record of these conversations, has increased for the most serious maternity incidents [29, 30, 32, 34]. However, little is known about which interventions, if any, have encouraged more frequent OD and how OD events are experienced by those involved. Accordingly, this realist synthesis of international evidence in maternity care was conducted to identify some of the critical factors that influence OD practices and outcomes that will later be ‘tested’ by in-depth national interviews and ethnographic case studies in a second phase of this NIHR-funded study [35].

This realist synthesis aimed to understand, as far as possible, how, for whom, why, and under what circumstances interventions designed to enhance OD influence these events and the experience of these events in maternity care. The research question guiding the synthesis was: ‘what key factors (resources and relationships) underpin the OD of incidents of harm in maternity care with affected families and how do they shape the expectation and effects of OD for different social groups—families, clinicians, and managers of services—in different circumstances?’ In all, our focus on OD improvements in maternity services is expected to encapsulate key issues arising in OD interventions in healthcare more generally. The review also aims to surface the contexts and effects of OD in various clinical situations or services where the aftermath of an incident is particularly complex and emotionally laden.

## Approach and methods

There are a variety of methods used to inform realist reviews, evaluations, and syntheses, however, all seek to explore how a programme, intervention, service, or policy works for different people and in different contexts. Using this approach, it is assumed that it is possible to identify a series of ‘mechanisms’ or ‘underlying factors’ that, when ‘triggered’ in particular contexts, set in motion different effects. These mechanisms include material elements (resources, constraints, and opportunities) and social-relational elements (the reasons and responses of people). Depending on the context, mechanisms might directly or indirectly influence or compete with each other in ways that can cause unintended outcomes [36]. A Context-Mechanism-Outcome (C-M–O) heuristic guides the identification and theorisation of how an intervention can have certain effects within specific conditions [37]. Table 1 briefly summarises the realist terms and techniques used in this paper and provides illustrations of these terms using examples from Waldron et al.’s (2020) paper on shared decision-making (SDM) [38].

**Table 1:** Definition of realist terms used in this realist synthesis

**Programme Theory**
The often hidden assumptions about how an intervention works [37] that are contained within the literature on the intervention, for example, the assumptions of programme designers [39]. These are first identified from the literature as a series of ‘if…then…’ or explanatory accounts (EAs) [38] that are more or less explicit theories about what ‘what creates change’ [40]. These might later be ‘tested,’ developed, or overturned by primary research findings
**Context**
Situations and settings that ‘trigger’ particular mechanisms [41]. For example, Waldron et. al (2020) identify three significant contexts (pre-existing relationship; difficulty with decision; health system support) for SDM and identify these as impacting on all mechanisms. Their example highlights the practical limitations of available literature, albeit with stakeholder discussion [38]
**Mechanism**
Resources and relationships that produce a particular effect [42]. There are likely to be multiple and sometimes competing mechanisms within a single intervention [40]. No single study can identify all mechanisms or all aspects of a mechanism [36]. For example, anxiety, trust, perception of time, and self-efficacy are identified as key mechanism sets for SDM [38]
**Outcomes**
Effects of a mechanism that can be immediate or longer-term, of varying depth or duration, and impact on particular social groups in particular ways [38]. They might also be a conceptualised as a single outcome of a programme theory [38] or understood in terms of multiple, fluctuating outcomes [43]

Realist literature syntheses seek to identify C-M-Os from within the data available in a document and not only from description of ‘research results.’ Examination of the ways that data is used and discussed in documents is expected to surface working hypotheses – or ‘initial programme theories’ – in relation to C-M-Os. That is, to identify ideas within a data set or document about how change happens, for whom, how, in what circumstances, and why [36].

A realist technique for surfacing initial programme theories from data is to extract them as a series of EAs for the included documents. Realist studies, like other forms of evidence synthesis, involve non-researcher contributors with subject or experiential expertise as collaborators in identifying and theorising [44, 45]. As are considered with these expert stakeholders for organisation, abstraction, and prioritisation to develop a manageable series of middle-range theories. Middle-range theories in realist analysis have been described by Emmel as “bundles of hypotheses that can be tested empirically” [46]. Testing is possible because these theories are abstract and can therefore be applied across cases that are empirically diverse. The following sections will describe our application of this approach to the realist synthesis.

### Search strategy

The documents included in the synthesis were identified using a two-stage literature search.

Stage 1 of the literature search, which took place in early 2019, conducted by authors MA and JH, involved a scoping search of the literature. The purpose of this search was to establish an overview of available international interventions for OD improvement (national, organisational, and individual/team-based). Search terms were developed in consultation with a subject specialist to ensure identification of relevant key words, synonyms, and spelling variations. A search term strategy was developed for MEDLINE (OVIDSP) and adapted for the other databases, CINAHL, HMIC, MEDLINE, PsycINFO, and EMBASE. These databases were selected to ensure comprehensive coverage of medical, nursing, psychological, health service policy, and social science literatures. An example of the MEDLINE database search was disclos*.mp AND adverse event*.mp (mp = title, abstract, original title, name of substance word, subject heading word, keyword heading word; protocol supplementary concept word, keyword heading word, unique identifier). This is presented in more detail in Additional file 1: Appendix 1. All sources that were published or translated into English and published after the year 2000 were included. Sources published prior to the year 2000 were excluded, as these pre-date the patient safety movement becoming significant internationally [47]. Following guidance on realist data gathering [45], no pre-determined exclusion criteria on research methods were applied. Grey literature, including policy reports, service guidance, and pubic and professional commentary were retrieved using free text searches in the Grey Literature databases (OpenGrey; OpenSource; Google Scholar). We also conducted free-text searches of Proquest and British Library EThOS Thesis records. Citation searches and reference list snowballing of included studies supplemented the database searches. All records were pooled into a bibliographic database and screened to exclude duplicate entries. Without duplicates, 993 sources were identified. For quality assurance, Medline, CINAHL, and Proquest searches were repeated in August 2019 with no additional papers identified for inclusion.

Stage 2 of the literature search was conducted between August 2019 and January 2020 by authors MA and JH. The purpose of this search was to identify, from our bibliographic database of 993 sources, data or documents on interventions for OD improvement in maternity policy, organisations, programmes, professions, and teams. This two-stage search strategy enabled us to identify papers that included analysis of organisational and national interventions that explicitly included maternity service areas, but that may have been missed by exclusively using maternity and disclosure search terms [6, 48–54]. The search of our pooled database involved a free-text search of complete documents (title, abstract, full paper, and key words) for terms identified by an additional subject specialist in maternity services. Terms searched were: matern*; obstetric*; midwife*; perinatal*; and childbirth.

Following realist guidance, the selection of document and data was expected to evolve in relation to the suitability of sources for addressing the research question [55]. The approach to final document identification was revised twice in ongoing consultation with five co-investigators with different subject expertise (see details below). First, it was agreed that only documents that either contained primary data or were systematic reviews ought to be included. This was because researchers identified many position papers arguing for the benefits of OD in maternity services with no evidence of implementation strategies or outcomes. Second, given increasing policy interest in OD in UK maternity services from 2015, it was agreed that reports on progress and outcomes from OD interventions from 2000–2021 should be screened for inclusion. These were identified by co-investigators who were subject experts (RI and AH).

### Literature appraisal

Next, identified documents were appraised for ‘fitness for purpose,’ that is, for their potential to contribute to our synthesis based on their relevance and rigour [42]. To assess relevance, or their potential to contribute to theory-building or theory-testing [55], we tailored an appraisal tool using the Critical Appraisal Skills Programme Checklist (CASP) (add link here). Data were appraised by two researchers (MA and JH) and ranked based on their potential to surface C-M–O elements (with 1 = highest ranking and 5 = lowest ranking). To assess rigour, or the credibility of the data based on the methods used to generate it, we tailored an assessment tool based on existing principles of research rigour [56]. Data and documents were assessed by MA, with documents ranked based on their credibility with respect to validity, reliability, and generalisability of findings (with 1 = all components included and 5 = no components included). We used theoretical definitions of these components [37, 57] to clarify the application of the tool to the qualitative and grey literature (for further details, see Additional file 2: Appendix 2). Given the purpose of the realist synthesis, documents with primary data on outcomes scored higher in appraisal ranking.

### Data extraction

**Fig. 1:**
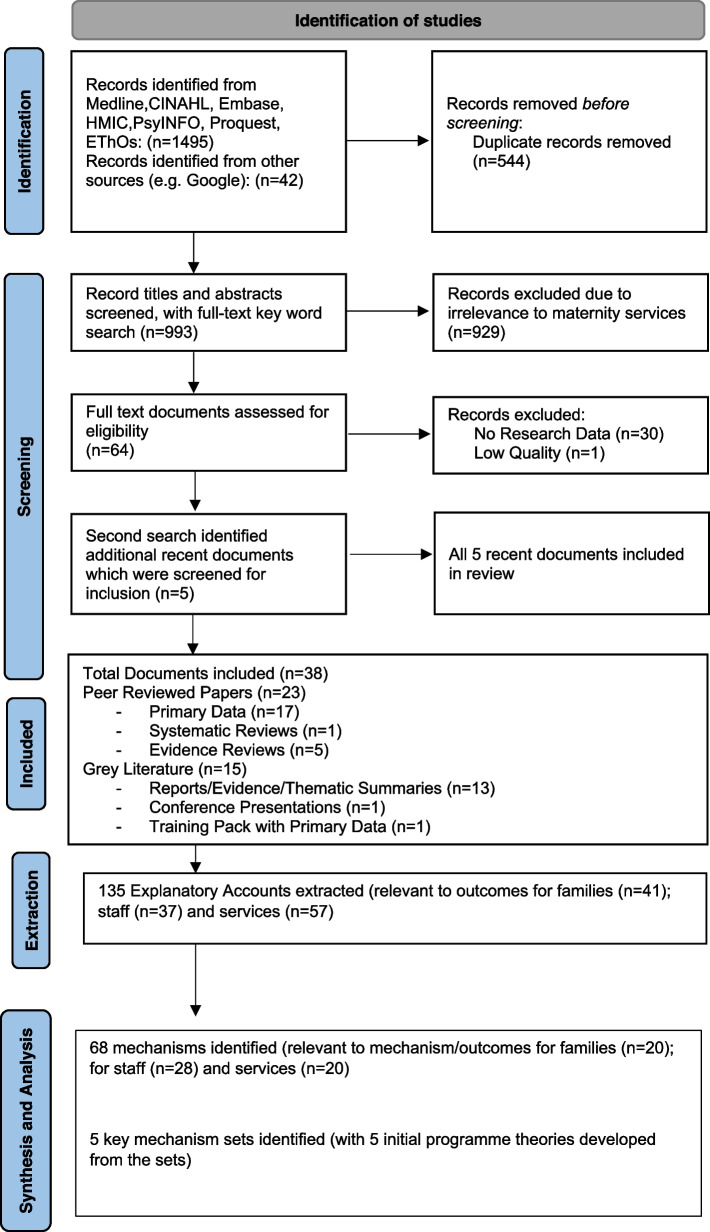
Screening and Synthesis Process. This figure depicts the process undertaken to reach the final five key mechanisms

The purpose of data extraction was to identify significant features that shaped and underpinned the effects of the improvement work and the contexts in which these are triggered. After a full reading of each document, researchers identified the EAs in each document. In line with the realist approach, these rationales were identified as sets of “if…., then….” propositions and, if possible, any propositions about this if/then connection were noted. A structured template that included bibliographic information, country of research, explicit or implicit rationales (with illustrative quotations), and reflective notes on emergent programme theories was developed and piloted for data extraction by the research team. It was anticipated that EAs would include taken-for-granted assumptions about ‘what works, for who, and why’ and so would extend beyond the primary focus of the study. Multiple EAs might also be embedded in single statements. Figure 1 depicts the screening and synthesis process undertaken to reach the final five key mechanisms.

### Stakeholder consultation

Collaboration with expert advisors happened three times over the course of the synthesis and with two different stakeholder groups. These were, first, the independent project advisory group (PAG) and the study co-investigator group (CIG). Both groups were composed of subject experts from a range of policy, clinical, patient and public interest, and/or research backgrounds.

### Stakeholder Consultation (1): The Project Advisory Group

Initial findings from data extraction were presented to 14 members of the PAG at a face-to-face, semi-structured, three-hour meeting in November 2019. PAG members were identified and invited to this meeting because they were already directly involved in OD improvement work, as policy makers and/or clinical leads (*n* = 4); third-sector leads (*n* = 2); legal experts (defense and claims) (*n* = 4); or as families (*n* = 4) working in educational or safety improvement advisory roles because they had been previously impacted by poor or successful OD practices. Professionals and families were identified and invited through national third-sector or health organisation networks. Following the advice of our university ethics committee, ethical approval was not sought to include these families in the meeting, because they were recruited as subject advisors, and not as research participants. However, the researchers followed a protocol for supporting families, reminding them that they could withdraw at any time, contacting each individual at the close of each meeting to ensure that no distress had been caused, and, if required, offering them access to specialist support provided by our third-sector agencies. The purpose of PAG consultation was to explore the focus and range of the included data and to seek advice on the relevance of emergent findings. Detailed minutes were kept of the meeting, that were later circulated to PAG members for agreement.

### Stakeholder Consulatations (2): Study Co-Investigators

Six subject experts who were also study co-investigators met vitually or face-to-face four to six weekly and advised on ongoing data extraction and synthesis. Their backgrounds were obstetrics (AH); maternity policy, midwifery, and social science (JS); patient safety and communication studies (RI); birth trauma support (MT); stillbirth and neonatal death support (CB); and birth rights (MB). Minutes were kept of their ongoing advice on document searching and inclusion, emergent data analysis and synthesis, EA consolidation, prioristisation of identified mechanisms in relation to the research question, and write-up.

## Results

### Document selection and appraisal

Nine hundred and ninety-three sources were identified in Stage 1 of the literature search. These were compiled in a bibliographic database. In Stage 2, 64 documents were selected for further review. Thirty documents that did not meet the Stage 2 inclusion criteria (to have primary data or be systematic reviews) and were then excluded, leaving 34 documents to be included in the synthesis. A further five documents that met Stage 1 and Stage 2 inclusion criteria were identified by the PAG and the CIG during 2020 and were subsequently included in the synthesis. In total, 39 documents were appraised for ‘fitness for purpose.’ After quality appraisal, one document was excluded from the review due to lack of rigour. In total, 38 documents were included in the realist synthesis. The focus, national context, aims and objectives, research design and specified improvement/intervention documented in the 38 documents is reported in Table 2. Table 2 also reports the quality appraisal scores (ranking for relevance and rigour) for each document.

**Table 2:** Overview of the 38 documents included in the realist synthesis

Focus of Improvement	Lead Author & Publication Year	National Context	Publication type	Ranking (Relevance)	Ranking (Rigour)	Aims and objectives	Research Design	Improvement/Intervention Specified
Family-Clinician Relations and Care Provision	Bakhbakhi D. (2017) [58, 59]	High-Income countries	Peer- reviewed Research	1	1	Review of latest published research, guidelines, and best practice points	Evidence Review	Stillbirth Bereavement Care
	Ellis A. (2016) [60]	Western High-Income Countries	Peer-reviewed Research	2	1	Synthesis and meta synthesis of parents’ and healthcare workers’ experiences of maternity bereavement care in hospital settings	Systematic Literature Review	Practical learning points to improve research, training and ultimately care of parents who experience late stillbirth (> 24 weeks)
	Downe S. (2013) [61]	UK	Peer-reviewed Research	2	2	Analysis of parents’ experiences and views of interactions with hospital staff after perinatal death	Qualitative	Care of parents after perinatal bereavement
	Heazell A. (2013) [62]	International	Conference Proceeding Report	2	2	Evidence-based summary of international conference proceedings	Evidence Review	Bereavement support after stillbirth
	Make Births Better (2020) [63]	UK	Research Report	2	2	Findings on reported access to support after a difficult birth experience. Findings on professional training and service provision for this support	Survey	Birth Trauma Care and Support for Women, Families and Professionals
	Redshaw M. (2014) [64]	UK	Research Report	3	2	Investigation of parents’ experiences of care after stillbirth or death of their baby after birth, including offering and information of post-mortem and professional support to understand the report	National Survey	Bereavement care after stillbirth or death of a baby after birth
	Stanford S. (2016) [65]	England	Peer-reviewed Article	1	1	Narrative account of experience of harmful event during maternity care; difficulties with communication and outcomes for women and family	Qualitative	Communication and candour issues, women’s story, and response by a professional college
Clinical Skills, Training and Post-Incident Support	Bonnema R.A. (2009) [66]	USA	Peer-reviewed Research	3	1	Post-intervention study of pilot training intervention to evaluate effectiveness of ‘Being Open’ training	Survey	‘Being Open’ and Breaking Bad News graduate training
	Coughlan B. (2017) [67]	Europe	Peer-reviewed Research	2	1	Narrative review of phenomenon of ‘second victims’ and remediation systems in maternity services	Evidence Review	‘Second Victims’ of avoidable adverse events in maternity care
	Karkowsky C.E. (2016) [68]	USA	Peer-reviewed Research	3	1	Assessment of trainee-assessed effectiveness of simulation training for breaking bad news situations in obstetrics	Randomised prospective trial	Simulation training with obstetric residents
	Raemer D.B. (2016) [69]	USA	Peer-reviewed Research	1	1	Testing of best practice guideline for disclosure and apology to improve communication performance	Randomised Trial	Mixed-realism simulation
Perinatal Mortality Review (Development & Evaluation)	Bakhbakhi D. (2017b) [59]	England	Peer-reviewed Research	1	1	Analysis of bereaved parents’ views on involvement in the perinatal mortality review process	Qualitative	Parents' Active Role and ENgagement in The review of their Stillbirth/perinatal death (PARENTS) perinatal mortality review design portfolio
	Bakhbakhi D. (2018) [70]	England	Peer-reviewed Research	1	1	Exploration of healthcare professionals’ views on acceptability of and support for parent engagement in the perinatal mortality review process	Qualitative	PARENTS perinatal mortality review design portfolio
	Bakhbakhi D. (2019) [71]	England	Peer-reviewed Research	1	1	Development of core principles and recommendations for parental engagement in Perinatal Mortality Review Tool	Qualitative	PARENTS perinatal mortality review design portfolio
	Boyle et al. (2021) [72]	High-income countries	Peer-reviewed Research	2	1	Investigation of perinatal morality review meeting practices, including the extent of parent engagement, as reported by healthcare professionals in six countries	Survey	Perinatal mortality review meetings
	Burden C.B. (2018) [73]	England	Report	2	2	Summary of evidence-based policy recommendations arising from the PARENTS studies	Evidence Summary	PARENTS perinatal mortality review design portfolio
	Chepkin S. (2019) [30]	England	Research Report	1	2	First annual report on progress of implementation of the perinatal morality review tool	Thematic Review	Perinatal Morality Review Tool Progress Report
	Kurinczick J.J. (2020) [34]	England	Progress Report	1	2	Second annual report of progress of the national perinatal morality review tool	Thematic review	Perinatal Morality Review Tool Progress Report
	Sauvegrain P. (2020) [74]	France	Peer-reviewed Research	1	1	Examination of effects of implementation of mother’s inclusion in perinatal mortality audit interviews	Mixed methods	District-level Perinatal Mortality Audit
Organisation or Service Level Pilots & Evaluations	Bennett J.B. (2016) [75]	Scotland	Conference Presentation	1	3	Summary of principles, requirements, and initial outcomes of the ‘Being Open’ project (for scalability of training package)	Progress Summary	‘Being Open’ Scotland
	Gluyas H. (2011) [76]	Australia	Peer-reviewed Research	2	1	Case study of hospital-level changes following an inquiry to review the quality of obstetric and gynaecological services	Qualitative	Clinical Governance
	Healthcare Improvement Scotland (2016) [77]	Scotland	Resources with evidence of effect	1	2	Checklists, resources, and outcomes evidence developed for ‘Being Open’ pilot	Qualitative	‘Being Open’ training and staff support pilot resource
	Hendrich A. (2014) [78]	USA	Peer-reviewed Research	1	1	Case study of implementation of full disclosure protocol in 5 pilot sites (one organisation)	Mixed methods	Labour and delivery units
	Pillinger J.P. (2016) [53]	Ireland	Research Report	1	2	Process evaluation of implementation of open disclosure pilot programme piloted in 2 acute hospitals (including maternity units)	Qualitative	Trust Pilot Schemes
	Santos P. (2015) [79]	USA	Peer-reviewed Research	2	1	Evaluation of a multi-faceted model for managing malpractice in obstetrics, including a disclosure programme	Qualitative	Disclosure Programme
	Scholefield H. (2007) [49]	England	Peer-reviewed research	1	1	Organisational case study of improvement in quality and risk management processes in obstetrics, including parent involvement in adverse events	Document analysis	Internal Trust Investigations/Local Review
National and Regional Interventions, Evaluations & Audits	Care Quality Commission (2016) [50]	England	Research Report	1	2	Review of processes and systems in NHS Trusts in England on how NHS trusts identify, investigate, and learn from the deaths of people under their care	Mixed methods	NHS Trust Investigations and Reviews of deaths of patients (including maternity units) Local Review
	Care Quality Commission (2019) [51]	England	Research Report	1	2	Review progress and examples of good practice in implementation of the learning from deaths guidance	Qualitative	Learning from Deaths guidance implementation
	Health Safety Investigation Branch (2020) [80]	England	Progress Report	1	2	Report on progress of engagement of families in independent investigations	Survey	Family involvement in external investigations of serious incidents (including maternity incidents)
	Iedema R.A. (2008a) [6]	Australia	Peer-reviewed Research	1	1	Determination of which aspects of open disclosure ‘work’ for patients and healthcare staff (including maternity services)	Qualitative	Australian Open Disclosure pilot
	Iedema R.A. (2008b) [54]	Australia	Peer-reviewed Research	1	1	Exploration of patients’ and family perceptions of Open Disclosure of adverse events that occurred during their health care (including maternity care)	Qualitative	Australian Open Disclosure pilot
	Kenyon S. (2017) [29]	England	Research Report	2	2	Examination of local reviews of a random selection of eligible cases reported to the perinatal confidential enquiry on inter-partum and intra-partum related neonatal death, including parent notification and involvement	Thematic review	Trust-based local reviews of inter-partum and intra-partum related neonatal death
	Magro, M. (2017) [31]	England	Research Report	1	2	Thematic review of NHSR data to identify the clinical and non-clinical themes from cerebral palsy claim records that resulted in claim compensation and to highlight areas for shared learning and improvement, including family involvement in serious incident reviews	Thematic Review	Serious incident Investigation summaries submitted to NIHR for progression of cerebral palsy claim
	NHS Improvement (2018) [52]	England	Research Report	1	2	National consultation (of patients, families, the public, commissioners, providers, and professional bodies) on factors affecting serious incident investigations (including maternity) in NHS Trusts	Mixed methods	Serious Incident Framework Implementation
	NHS Resolution (2019) [17]	England	Progress Report	1	2	Analysis of a pragmatic sample of cases of potentially severe brain injured babies reported into year 1 of the Early Notification Scheme, including notification and communication with families	Mixed-Methods Thematic Review	Early Notification Scheme progress report
	Quinn A.M. (2008) [81]	USA	Peer-reviewed Research	2	1	Description of origins and outcomes of 3Rs programme for patients, physicians, and programme officers (including maternity)	Qualitative	The 3Rs programme (early disclosure and resolution program)
	Sakala C. (2013) [82]	USA	Peer-reviewed Research	3	1	Literature synthesis of policy strategies most likely to mitigate harmful effects of the liability (tort) system for families	Evidence Review	Liability Systems
	Sorensen R. (2008) [48]	Australia	Peer-reviewed Research	1	1	Analysis of views on open disclosure of medical errors by health care professionals and managers and identification of workforce and systems capabilities required for embedding disclosure in units	Qualitative	Australian Open Disclosure Pilot

### Issues raised at the PAG meeting and effects on the synthesis

**Fig. 2:**
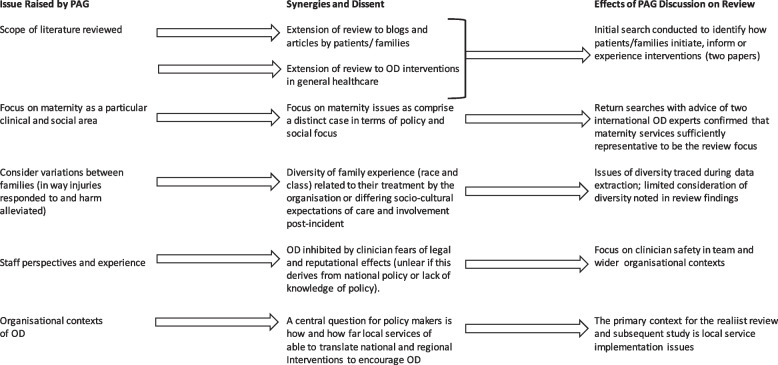
Visual Summary of the Effects of Project Advisory Group (PAG) Insights on Review

The PAG meeting advised on one query about document identification and raised and discussed a series of observations on the relevance of identified documents and emergent findings from them. The effects of Project Advisory Group insights on the synthesis (including issues raised, group synergies, dissent during discussion, and outcome) is represented as a visual summary in Fig. 2.

As Fig. 2 indicates, the PAG consultation did not influence change in the initial data extraction process. However, the consultation did influence data synthesis, as the PAG prioritised mechanisms operating at inter-organisational and team-level practices, rather than national drivers like regulations, policies, and programmes that might be assumed to be more obvious mechanisms for OD improvement. The PAG also directed the researchers to a more thorough consideration of the immediate and ongoing social and emotional effects of OD on healthcare staff and the relationship between this and OD outcomes.

### Characteristics and subject focus of the documents

The 38 included documents, organised by comparable interventions, publication details, realist quality appraisal ratings, and key study characteristics, are presented in Table 2. These include publications in peer-reviewed journals (*n* = 22); progress reports by organisations (*n* = 14); and evidence-based improvement updates with training resources (*n* = 2). One paper was a systematic review of international evidence and two papers were evidence reviews. The type of evidence reported in the papers was qualitative or qualitative data on self-reported or other-reported data on views and experiences of OD or OD improvement interventions. The documents included findings from England (*n* = 18), the USA (*n* = 7), Australia (*n* = 4), ‘High-Income Countries’ (sic) (*n* = 3), Scotland (*n* = 2), Ireland (*n* = 1), France (*n* = 1), Europe (*n* = 1), and ‘International’ (sic) (*n* = 1). The location of findings is notable because of differences in the policy and medico-legal contexts in which large-scale OD implementation policies are developed. For example, disclosure guidance and policies are most highly developed in the Commonwealth countries [22] and, from 2013 in England and Wales, a statutory ‘duty of candour’ has been required of health providers, a legal requirement that resembles US State apology laws (in 35 States) [22]. There is a complex relationship between national policy, broad litigation trends, and local policy and practice development. Wu et. al (2017) note the development of diverse and innovative disclosure programmes in the USA, where decentralised governance of health services and concerns with liability costs encourage individual institutional action rather than litigation [22].

Ten papers documented three significant research programmes: evaluations of outcomes of national improvement in Australia [6, 48, 83]; process and outcomes evaluation of organisational interventions to improve OD across five pilot US hospitals [78, 79]; and documentation of design and development of a perinatal mortality review in England [58, 59, 70, 71, 73]. Across all papers, there was limited primary research investigating families’ experiences of OD and what families consider necessary for OD in maternity services (except for Iedema [54], Quinn [81], and Stanford and Bogod [65]). The question of ‘what families want’ was more often assumed. Only two papers considered social diversity as a factor that might influence experiences of OD and felt outcomes [54, 60]. Evidence of the direct use of family experience for practice or systems change was limited to one paper [65]. While ‘culture change’ toward ‘fair’ or ‘no blame’ practices was often mentioned as an overarching cause [31, 32, 49, 50, 59, 81, 82] and/or effect [49, 50] of OD improvements, this was more often used as an overarching term, without a more nuanced approach to understanding aspects of change and variations in ‘virtuous circles’ [84].

The empirical studies and reports documenting the effects of OD interventions (*n* = 21) were overviewed for descriptions of intervention design and intervention outcome. These fell into three broad categories of intervention (Table 2) and the nature of the evidence on outcome across these studies was highly varied. First, three quantitative and mixed-methods studies examined the outcomes of simulated training sessions for individual trainees or professionals that were designed to enhance clinical communication skills (*n* = 3) [66, 68, 69]. These studies all suggested that there was an improvement in individual or team skills to conduct OD conversations after the interventions, with one identifying some of the benefits from the use of an evidence-based cognitive aid [69]. However, these clinical educational studies were small-scale (*n* = between 15 and 60 participants), conducted in simulated environments, and most significantly, did not include patients or the public perspectives on the study design or assessments of outcomes.

The second group of studies included four progress reports and one qualitative study, which all documented the progress of parent or patient involvement in safety improvement interventions. These included consideration of perinatal mortality reviews or audits (*n* = 3) [30, 34, 74] and serious incident investigations (*n* = 2) [50, 51]. These studies indicated the slow progress in making improvements around when parent participation is introduced as one element of a wider national safety improvement initiative. Issues of capacity, capability, and attitudes of staff working in services that engage with families are not the focus of these interventions, although these are known to contribute to the slow pace of engagement work.

Third, a series of studies and reports (*n* = 8) documented the effects of multi-faceted interventions to strengthen OD practices organised across a sector, service, or hospital [6, 48, 54, 75–78, 81]. These interventions were often described as including the development and dissemination of faculty-tailored protocols and guidance, formation of clinical governance revisions, and introduction of general and more specialist HCP training, as well as wider awareness-raising across staff teams. Overall, these studies described or anticipated the long-term and uneven quality and extent of OD. They often captured the tension between clinicians’ support for OD in principle (and offered a few individual and positive experiences of the effects of honest apologies on clinician-patient relationships) and the wide-spread reticence of clinicians to risk the uncertain implications of OD to their or others’ reputations and the risk of the emotional impact for everyone involved in the incident. One study [78] was an exception in that it described a widespread increase in OD practices in one hospital-based on a quality assurance audit. The authors explained this quantitative change as a long-term (at least 27-month) consequence of dedicated resourcing and focus by senior leadership, consistent messaging throughout the organisation, investment in enthusiastic and established champions working close direct care provision, and insurer-approved protocols and specialist OD leads. However, with few exceptions [6, 81], the views and experiences of patients, families, and staff on the quality of OD events and their felt consequence was not a focus of these accounts of service-based OD improvements.

Overall, the identified documents described a variety of interventions intended to improve and evaluate OD practice in different ways. They ranged from interventions targeted at individual clinician attitude or practice change, to revisions in particular systems for reporting and audit, and to state-wide or national interventions to enhance OD through policies of regulation, incentivisation or awareness-raising within provider organisations.

### Realist data extraction

As anticipated, identified EAs were not necessarily the primary study focus of the 38 selected papers [29]. EAs were extracted for three interest groups, families, staff, and services, and were reported separately for each group. Where mechanism/outcomes were documented for two or more interest groups, the EA was counted for each of the groups. In some sections of text, multiple EAs were identified in a single statement, and these were reported separately. 135 EAs were identified from the 38 documents, these included: EAs specific to families (*n* = 41); healthcare staff (*n* = 37); and services (*n* = 57). Across the 38 documents, we identified at least one C-M–O configuration from 34 papers, with 23 of these documents reporting evidenced outcomes, and 11 of these papers surmising likely outcomes (see also Table 2).

### Analysis and synthesis

Analysis of the extracted EAs was completed in five steps:

1. First, for each interest group (families, staff, and services), the researchers examined the EA statements to establish themes based on semi-predicable patterns in the statements [41].
2. Second, the EA statements were mapped across two pathways. These were (a) a pre-identified ‘ideal-type’ temporal trajectory of an OD processes (from event identification to resolution) [85–87]; and, (b) in relation to context/mechanism relationships identified for the EAs.
3. Third, these documents were shared with our Co-Investigator Group (CIG) so that agreement on consolidation and prioritisation could be reached (see below for more information on the CIG stakeholder group). The CIG prioritised EAs when: (a) it was agreed that they were likely to have a strong relationship to OD improvement (for example, patient access to medical records was excluded); (b) when more immediate or intermediate outcomes for family or staff groups were likely (for example, longer-term and surmised changes in social or public benefits or costs from OD improvement were excluded); and (c) when demi-regularities were felt to have some equivalence (for example, an open-door policy for family involvement and responsiveness to family needs with respect to the timing of their involvement were counted as the same). This synthesis resulted in the identification of 68 consolidated EA statements across the three interest groups: family (*n* = 20); staff (*n* = 28); and services (*n* = 20).
4. Fourth, the researchers organised these 68 consolidated EA statements thematically to identify C-M-Os, or elements of C-M-Os. This also included the identification of the resourcing and responses/relational aspects of identified mechanisms and the thematic analysis of contextual factors for each of the interest groups.
5. Finally, in a subsequent meeting with the CIG, the team identified and named five sets of mechanisms that they considered to have the most notable causal effects for OD (and so to be most critical to success).

Figure 2 describes the screening and synthesis process. The results of this EA thematic grouping and mapping exercise for each of the interest groups, along with the EA codings, for each group, are documented in Tables 3, 4, and 5.

**Table 3:** Explanatory accounts for improvements in open disclosure: what works, when and how from a family perspective (bolded explanatory accounts for services (eas) have been included in final c-m–o configurations (Table 6)

IDENTIFIED EXPLANATORY ACCOUNTS FROM THE 38 DOCUMENTS				
**IMPROVEMENTS IN OPEN DISCLOSURE: WHAT WORKS, WHEN, AND HOW FOR FAMILIES**				
**EXPLANATORY ACCOUNT FOR FAMILIES (EAfam) REFERENCE**	‘BEING OPEN’ PATHWAY	SITUATION	Indications of Mechanisms (forces, interactions, reasoning, and resources)	Outcomes for Parents/Family
**EAfam1**	EVENT IDENTIFICATION WITH FAMILY	Incident may be catastrophic or gradually identified; outcomes may be uncertain or develop over time (12 references)	Timely and reliable confirmation of incident [58]	Reduces prolonged anxiety [58]
**EAfam2**			**Ongoing and flexible identification of incident type/severity **[50, 63, 65]** in meetings and record-keeping **[50, 63, 65]**; follow professional duty of candour and incentivised schemes to promote candour **[48, 54] Routine invitation to family to discuss the felt incident pre-discharge/systematic assessment of reported symptoms [63]; standardised checks embedded across maternity care pathways [63]	**Shows respect for parents’ views and experiences **[50, 63, 65]**; promotes timely referrals **[63]** by ensuring that subsequent providers have information for care/referral account of incident to other providers **[63, 65]**; encourages services to engage with families **[48]; **may include disclosure of incidents with lower thresholds of severity **[48, 54]
EAfam3			Sensitive timing of news [61, 64]; partner involvement [60]; acknowledgment of religious and cultural preferences, language needs, and use of tools with informed guidance [62, 64] to enable decision-making for investigations (e.g. post-mortem) [58]	Seen as necessary for ongoing involvement [58]; reduces psychological demands [61]; enables best decision-making that helps later coping [60, 62, 64]
EAfam4			Co-ordinated communication with original provider/across facilities when an event is identified later in a different facility [6]	Reduces need for repeated explanation [6]
EAfam5			Uncomplicated and supported access to own health records and information [50]	Reduces suspicion that the service is hiding things behind ‘patient confidentiality’ [50]
**EAfam6**	**ONGOING CARE AFTER EVENT**	**When the incident has happened (7 references)**	**Positive interactions with healthcare staff via acknowledgment and prioritisation of the patient’s situation **[58, 60–62]**; reducing feelings of being ignored or having the event overlooked; emotional **[61]** and respectful care** [50]**; continuity/consistency of expert care **[58]** and information from all staff **[61]** required; information on how to navigate unexpected/unusual clinical situations **[61]	**Efforts are highly valued by families who are facing the unknown **[61]**; care needs are met **[58]**; reduces confusion/distress or felt/expressed frustration towards immediate care staff **[61]**; reduces sense of isolation, confusion, and vulnerability **[65]** and decreases long-term negative consequences of bereavement **[62]**; reduces loss of confidence in HCPs **[61]**; sets a positive tone at the start of reviews/investigations **[50]
**EAfam7**	DISCLOSURE PROCESS	Structures and Strategies (8 references)	**National guidance, mandates, and programmes drive and routinise formal disclosure procedures and translate these into clear unit policies to include: proactive family engagement; sensitivity to diversity and individual needs **[6, 59]**; prompt triggering for severe adverse events (various definitions) **[6, 54, 64, 77, 81, 82]**; possibility of consent to further investigations **[59]** and early discussion of review/investigation decisions **[51, 77]	**Avoids demands on family to’chase’ providers for information **[6, 54, 64, 82]**; changes their perception of events (‘self-preservation’ of service less often assumed) **[50]**; families feel treated as partners **[6, 51, 59]** (however these formal directives do not, in themselves ensure involvement of families in all events as regulations may be infrequently followed, e.g. definitions of severity may vary) **[77]
**EAfam8**		Service Ethos (3 references)	**Ongoing/established practices in an organisation that embed and sustain ‘taken for granted’ involvement **[72, 75]**; involvement/engagement reinforced by wider service/organisational practice and ethos **[34]	**Involvement becomes routine practice in incidents/situations **[34, 72, 75]
**EAfam9**		Service Governance (references)	**Representation of families via review/investigation committee membership **[72]**; service/Trust oversight of family involvement **[51]	**Sustains awareness of family in meetings **[72]**; increases a sense of family entitlement to involvement **[51]**; families are able to inform or oversee improvements **[51]
EAfam10			Commissioners are pro-active in investigation/action plan oversight [77]; Board-level responsibility for Candour regulations (and for inclusion of parents and staff in investigation processes) [48]; networked governance structures to enhance disclosure practices (Board-level, Membership Councils, QI Steering Groups; Patient Leads) [49]; annual reporting of national bodies to include lay summaries [71]	Ensures better involvement/candour [48, 77]; reduces variability of investigations [77]; embeds an expectation of family involvement in routine management [49]; engages public sector in quality improvement processes [71]
**EAfam11**		**Accessibility and Availability of Disclosure Process (12 references)**	**Routine and timely invitation for parents’ views, concerns, and questions after incident **[6, 34, 50, 54, 70]** (including what action to be taken) offered multiple times **[34, 64, 70]	**Reflects best practice as agreed by families **[6]**; reduces felt mistrust **[50] **(but invitation does not, in itself, result in parents asking questions) **[34, 70]**; gives time to reflect on events **[70]** and plan questions **[54]**; increases awareness of opportunities to be involved **[34]** and opportunities to return until the family feels less dissatisfied **[64] **(However, systematic and routine engagement practices are no guarantee of active participation **[72]**)**
**EAfam12**			**Family-centred/personalised approach to disclosure discussion/follow-up **[50, 59, 80]** with staff freely available to respond to variability **[54]**; including meeting specialist needs (e.g. language services) **[80]**; an open-door policy to when and how to contribute **[59]	**Decisions on degree and nature of involvement are possible **[50, 54, 59, 80]** and these rest with the family **[50]** or they have a voice in the process **[80]**; open-door policy may be retriggered in subsequent pregnancy **[59]
**EAfam13**			**Disclosure process explained **[52, 76]** in understandable way **[77]	**Leads to understandable information with minimal requirement of active involvement unless desired by family **[77]**; an opportunity for questions to be addressed **[76]**; the system feeling less ineffective or closed to families **[52, 76]**; decisions being made with people **[76]**. Reduces anxiety and confusion over accountability issues **[52]
**EAfam14**		Places Enacted (9 references)	**Booked meetings with families are formal and planned by lead clinicians **[54]**, with space and time for the parent, in a comfortable environment **[34, 54]	**Shows families that the event is taken seriously; responses to questions are considered/more reliable **[54]**; families feel more able to prepare to raise questions and concerns **[34]
**EAfam15**			**Conducted (ideally face-to-face) with nominated clinical expert **[64, 73]**, with awareness of family situation **[60]**; or with those originally involved in care **[71]** (or with further opportunity to meet with them) **[54]	**Reflects agreed best practice by parents **[73]**; provides emotional support **[60]** and chances to ask questions and discuss events directly **[71]** (and not just as a recipient of information **[64]**); shows respect for personal situation **[54]
EAfam16			Exclusion of legal and external/ ‘arms-length’ presence at meetings [50, 81]	Increases direct communication of family with clinicians [81]; feels less intimidating [50]; increases trust; tensions are reduced [50] (legal advice to providers should be on meeting candour and patient involvement principles) [50]
**EAfam17**		Early Disclosure Conversations (12 references)	**Staff skilled in active listening **[6]**; using ‘carefully chosen words’; aware of effects of language **[75]**, posture, and conversational tone **[69]**; attuned to the family’s experience **[54]** (responsive to expressed needs and cultural preferences **[6]**)**	**Seen as a crucial aspect of effective disclosure **[6, 69, 75]** that can lessen harm **[54]**. Improves human communication by health professionals, with the most significant change felt by patients **[76]
**EAfam18**			**Authentic **[67]**, honest and direct **[6, 51, 62]**, and timely apology **[65]** (uninhibited by felt litigation risk **[51, 81]**; and with the provision of a ‘safe space’ **[50]**)**	**Maintains trust in clinician **[67]** or service**^6^**; is valued by some parents because it is empathic **[62]**/suggests partnership working with them **[51]**; can avoid damage to healthcare relationships **[81]**; and enables openness after mistakes **[50]
**EAfam19**		Explanations (5 references)	**Initial clarifications that not all investigations establish cause **[58]**; reviews/investigations might not answer all questions **[80]**; findings may be inconsistent across multiple investigations of same event **[50]**; focusing may focus on systems-change and not individual cases **[80]	**Reduces disappointment, distress **[58]** and mistrust **[50]**; may facilitate helpful signposting to additional information or organisations **[80]**; the identification of an accountable person might be expected by a family **[80]
**EAfam20**			**Exploring initial expectations: local review of care (including avoidability and future care issues) **[34, 62, 64]	**Local reviews (event and findings) are a critical/’life shaping event’ for many **[34, 62, 64]**. Families expect information on why (explaining past; planning future) and/or systems-wide improvement **[71]
EAfam21		Consistency in Disclosure Process (7 references)		Improves the consistency of care and information [54, 58, 60]; leads to fewer staff asking the same questions [6]; shows that the event is not minimised or quickly forgotten [54]; provides opportunity for irreconcilable views to be explored [54]
**EAfam22**			**Information-giving through course of multiple investigations (for same event for different purposes **[50]**); future possibility of a single, integrated report **[34]**; clarification of ‘investigation hierarchy’ **[52]	**Reduces inconsistency/ experience of un-coordinated services **[50]**; avoids contradictory information and advice **[34]**; reduces felt disagreement **[52]
**EAfam23**		Navigation of Disclosure Process (10 references)	**Named contact people for ongoing family support **[6, 73]**, liaison, or advocacy from initial disclosure to inquest **[50–52, 71, 73, 80]**; continuity of contact where possible **[80]**; follow-on support arranged before discharge **[51]	**Agreed best practice by families **[6, 51, 73]**; positive effect on the experience of families overall **[52]**; supports ongoing **[50]**, flexible, and diverse **[80]** involvement (including family feedback on investigation process) **[73] **Note:[however stakeholders not agreed on if this liaison personnel or advocate should be independent of, or embedded in, investigating or clinical service] **[71]
**EAfam24**			**Family nominated advocate or HCP (such as bereavement midwife) to attend review meeting; ask questions on family’s behalf **[72, 73]**; explain particular circumstances in that review/investigation (e.g. delays) **[82]	**Leads to family representation **[72, 73]**; information-giving and reassurance to families on progress of progress **[52, 73]**;** **advocacy relationship might diffuse family anger and harm resulting from event or poor or delayed investigation process **[50, 82]
EAfam25			Joined-up systems (PALS, complaints, incidents) [51]	Reduces points-of-contact for families [51]
**EAfam26**	DISCLOSURE DURING REVIEWS AND INVESTIGATIONS	When incident review and/or investigation initiated (24 references)	**Family pro-actively included in decisions on review/investigation from outset **[34, 50, 51, 77]**; able to raise nonclinical range of questions and opinions; perspectives and comments accommodated (and independent investigator ‘checks’ this opinion-seeking has happened) **[50]**; centrality of family views embedded in review/investigation process **[70]** and process design **[59, 70, 71, 73]	**Inclusion of family experience and perspectives **[50, 59]** means that investigations or reviews more meaningful **[34, 77]** and effective **[77]** for the family (however est. 59% of reports where questions of family not addressed) **[50]**. Reduces distrust; accuracy and credibility of investigation are enhanced **[50, 51]**; involvement in finding explanations may alleviate harm **[70]**; engagement could be extended to other services **[73]
EAfam27			Use of nationally agreed standards [77], with policies and local guidance with co-ordinated, consistent, and explicit rationale and approach for parent involvement [50, 72, 77]; standardised mortality review tools incorporate family involvement [34, 70, 71] standardised communication process (that allows tracking of progress) [77]	Reduces variation in involvement across cases and units [50, 51]; involvement more central to investigations/ investigation quality assessment [77]; more co-ordinated and consistent communication possible [77]; More likely to be informed of review and invited to raise questions, concerns [34, 70, 71] (concerns/questions raised by 58% of parents) NB (policies do not necessarily guarantee respectful and caring family involvement [60])
**EAfam28**			**Comprehensive reviews/investigations include whole care pathways **[34, 58, 59]** with multi-disciplinary/cross-service representation **[76] **with families and subsequent sharing of knowledge of events/effects beyond that service **[65]	**Incorporates overall family experience of care **[58, 76]**; prevents loss of information **[76]**; could avoid further investigations with costs to family **[34]**; enhances learning for system-improvements **[34, 59]** encourages wider service responsiveness to recommendations for ongoing or subsequent care requirements **[65]
**EAfam29**			**Structured and accessible general information for families on steps and timescales of review/investigation with family-centred design and delivery **[48, 52, 58, 60, 70, 77, 82]	**Minimal requirement for family’s active involvement if they choose **[77]**. Family more likely to be included in the process **[52, 60]**; decision-making **[58]**; ability to ask questions **[70]**; and understanding reasons for investigation **[48]** or time it may take **[50, 82]
EAfam30			Clarification of the primary objective of that review/investigation for a family [80]	Reduces misunderstanding and disappointment [80]; directs appropriate questions and defines expected limitations of review [80] NB (however families sometimes anticipate that review multiple purposes, from explaining what happened [34, 61] to recommendations for wider learning and prevention [61])
**EAfam31**			**Specialist (emotional and practical) support and advocacy provision for families (and information on this) **[50, 77]**; user-groups advise on least harmful timings/approaches to family **[73]	**Necessary if families to be included in investigations **[77]**; agreed best practice **[73]
**EAfam32**			**Individualised/flexible or ‘open door’ opportunities for Involvement **[51, 59] **that are appropriately timed **[54]**, high-quality review/investigation process (contribution to ToR, questions and report drafts) **[34, 50, 75, 77, 80]**; with named support of, and formal documentation of, parent feedback on this process **[73]	**Accommodates individual and changing needs **[51, 59]**; best practice principles (as agreed by parent representatives **[73]**);or expectation of active involvement **[34, 77]**. Families are more likely to be involved in and satisfied with report **[50, 80]**; there is an appreciation of honesty, openness, and detail **[75]
**EAfam33**			**Meaningful apology and explanation to family for avoidable harm **[48–50, 77]** (that is timely **[65]**) with assurances of learning **[48, 49]**; expression of regret from those accountable **[48, 54]	**Necessary recognition of the familiy **[77]** and accountability **[48]**; trauma may be reduced **[49]**; personal resolution possible **[54]**; trust in health care provision might be sustained **[48]**; and the situation is less likely to escalate to complaint about concerns or legal action to get answers **[49, 50]**. However, when apologies are offered too late (or the family are not ready to engage), trauma may be increased **[65]
**EAfam34**	OUTCOMES OF DISCLOSURE PROCESS	Reporting and Feedback (9 references)	**Informing /discussing with families as review/investigation continues **[52, 71]** (including delays) **[50]**, as well as discussion of final report findings and feedback on involvement process **[50, 52, 79, 80]	**Prevents mistrust caused by either ‘closed door’ investigation and denial of ongoing discussion **[71]**; enables family concerns to be raised over time **[52]**; lessens information ‘drip feed’ (without possibility to ask questions) **[50, 79]**; final report more likely to be satisfactory **[50] **NB: (however: 24% of respondents agreed with value of family feedback survey for ongoing quality improvement (may be onerous from families and should be optional) **[52]**)**
**EAfam35**			**Reports are accurate, appear complete and without jargon **[50, 77]**; (if external) are forwarded to families before Trusts **[77, 80]	**Indicates that report is reliable, understandable **[50, 77], **and open from a family perspective **[80]
**EAfam36**		System-Wide/QI Revisions (8 references)	**Action (and accountability for this action) from review/investigation to prevent same event happening again **[5, 6, 50, 59, 81]**; selective in-depth investigations (including near-misses) to maximise learning **[52] **Leading/initiating change based on event/experience **[50, 65]	**Families want this to make sense of loss **[50, 59, 81] **NB: (however 83% families think that investigation had made no positive difference; 73% unclear on what learning had happened) **[50]**; some families want personal accountability for events **[80]**; exclusion of family’s own case from improvement programme might not be acceptable to them **[52] **Leading/assuring change may be adequate in some situations **[50, 65]
**EAfam37**		**Family Resolutions (3 references)**	**Offer of fair compensation (if admission of fault) **[82] **and payment of expenses/further access to services of involvement in disclosure process in all situations **[48, 81]	**Appreciated by families **[81]**; may promote some family’s involvement in disclosure processes **[48]**; diffuses anger and may preserve relationships **[82]
EAfam38		Indirect Social Revisions (7 references)	Public awareness (and information) on rights to raise concerns and to support/advocacy after incidents [50, 51]	Increases number of families informed/engaging [50]; decreases marginalisation after incident [51]
EAfam39			Revisions in clinicians’ awareness of effects of professional cultures on involvement and care [76]	Main barrier to involvement reduced for some, especially when more vulnerable and making decisions about involvement [76]
EAfam40			Improvements in communication skills of doctors [65]	Increases ability to deliver care more generally [65]
EAfam41			Wider awareness of value of family/patient insights along with clinical insights [50, 52, 80]	Recognition possible; reduces antagonism [50]; improves understanding of events [80]; view of families as disruptive is less likely [52]

**Table 4:** Explanatory accounts for improvements in open disclosure: what works, when and how from a staff perspective (bolded explanatory accounts for services (eas) have been included in final c-m–o configurations (Table 6)

IDENTIFIED EXPLANATORY ACCOUNTS FROM THE 38 DOCUMENTS				
IMPROVEMENTS IN OPEN DISCLOSURE: WHAT WORKS, WHEN AND HOW FOR STAFF				
EXPLANATORY ACCOUNT FOR STAFF (EAsf) REFERENCE	‘BEING OPEN’ PATHWAY	SITUATION	Indications of Mechanisms (forces, interactions, reasoning, and resources)	OUTCOMES for Staff
**EAsf1**	EVENT IDENTIFICATION	Incident may be catastrophic or gradually identified; outcomes may be uncertain or develop over time (6 references)	**Confidence in reporting systems (equity of response; learning from event); feedback on outcomes of incident reporting **[49, 50, 67]**; confidence in colleagues leading disclosure/investigations **[58]**; non-punitive reporting environment **[49, 52]**; systems for identifying good practice formalised **[49]	**Will increase confidence in when to trigger a formal response to an adverse event **[49, 50, 52, 67]**; with less anxiety over possible impact on reputation, relationships, and career **[58]
EAsf2			Recognition of different views of incident severity [50]	More frequent reporting of adverse events [50]
EAsf3			Protocols to support consistent decisions on when to investigate [50]; availability of decision-making tools for use with anxious/bereaved parents [58]	Will clarify expectations (including involving the family) [50]; the supported consent process will be less difficult [58]
**EAsf4**	**ONGOING CARE** **AFTER EVENT**	**When the incident has happened (4 references)**	**Capacity (resources, skills, behaviours, attitudes **[61]**) of staff to respond with emotional intelligence to needs/requests and choices of bereaved/traumatised parents **[58, 65, 69]** and sufficient opportunity to reinforce this across teams **[61]	**Reduces likelihood of expressions of anger and aggression toward staff **[61]**; staff more able to understand women’s requests **[58, 65]
**EAsf5**			**Availability of staff with specialist skills to share/model/disseminate responsive approaches to injured families**	**Leads to dissemination of skills and recognition of this work **[61]
**EAsf6**			**Provision of support during/after care of avoidable/unavoidable serious incident **[62]	**Reduces personal and emotional toll of the work (emotional difficulties that lead some clinicians to give up practice) **[62]
**EAsf7**	DISCLOSURE PROCESS	**Structures and Strategies (8 references)**	**Disclosure processes are supported and monitored by experienced colleagues **[58]**; are embedded in robust clinical governance systems **[58]**; and are provided by skilled staff **[51]** who have ongoing support, advice, and practical help **[52, 74]	**Reduces inherent uncertainties over disclosure practice (impact on own and organisational reputations or with a reduction in legal action by families searching for explanation **[58]**; disclosure practices by individuals is better supported **[51, 52, 74]**)**
**EAsf8**			**National mandate (Regulation 20) with ‘Being Open’ guidance **[51, 74]	**Emphasises organisational value of disclosure **[74]**; encourages organisational support for staff involved in this work **[51, 74]
**EAsf9**			**Collaborative Implementation of improvement work (e.g. new protocols) across service **[75]** or organisation **[78]**; demonstrated benefits of investment in specialist and senior support **[75, 78]	**Decreases uncertainty of clinical staff and managers and decreases resistance about changes in practice **[75]**/ they are less likely to resist **[78] **NB: (collaboratively developed protocol revisions over 80% more likely to be implemented **[78]**)**
**EAsf10**			**Educational programs and staff support are critical elements of disclosure programmes **[54, 77]	**Increases staff competence and relationship to involve family throughout process **[54, 77]
**EAsf11**			**Legally protected ‘safe spaces’ for disclosure conversations **[50]	**Decreases clinicians’ fear around legal consequences and increases the likelihood that families will learn the truth from clinicians; increases open relationship with a family **[50]
**EAsf12**		**Ethos (4 references)**	**Wider organisational landscape of trust between organisations and clinicians in which policies, tools, and programmes are operationalised **[61, 81]	**Engages clinicians in an ethos of early reporting and disclosure **[81]**; and improves positive relationships with injured patients **[61]
**EAsf13**			**Established practice that is supported consistently and clearly by local physicians and managers **[78]**; senior doctors role-modelled disclosure with patients **[67]	**Openness with families becomes ‘part of the mind set of all practitioners’ (about three months after initial implementation) **[78]**; medical students and junior doctors will aspire to emulate disclosure practice **[67]
**EAsf14**		Governance (3 references)	Implementation of disclosure widely supported by Trust leaders and managers (and government) [50, 73] and modelled by Trust leads[49]	Leads to the development of local cultures of reporting, openness, and learning [50]; reassures staff of this work[49] (and will not rely on a few champions [48]); promotes disclosure work as a clinical priority for services and teams [73]
EAsf15		Accessibility/Availability (5 references)	Managing parent expectations/questions (e.g. limited PMRT ‘free-text’) [71]	Services are able to manage questions in the time available for reporting [71] and to provide answers to the questions that families are asking [71] NB (however 50% stakeholders voted against time limit set for addressing parent questions in PMRT meeting) [71]
EAsf16			Inclusion in staff in review meeting schedules and invitations [49, 70, 82]; staff sensitively informed/kept informed of investigations involving them [52]	Staff are able to attend panel discussions that involve them [70] and feel less fearful and isolated during this time [52]
**EAsf17**		**Places Enacted (1 reference)**	**Time alone and with colleagues to prepare for disclosure conversation following a guide (who to contact; accommodating different understandings; what to say; body posture and proximity; how to respond; what is required) **[69]	**Equips staff to plan the conversation and follow-up **[69]** and leads to better conversations with families **[69]
**EAsf18**		**Initial Disclosure Conversations (13 references)**	**Communication training **[66, 75, 82]** for staff to acquire necessary interactional skills for difficult conversations **[82]**; training offered to all labour and delivery clinicians **[78]** and as part of the trainee curriculum **[66]**, including multi-disciplinary training to prepare for the disclosure conversation **[69]	**Staff who attend have increased skills and confidence **[66, 75]**and greater willingness to be involved in discussions with families **[68, 82]**. Their levels of stress and risk of burn-out are reduced **[68] **notably with all team approaches **[66, 78]**. Staff might also develop wider collaborative relationships **[69]
**EAsf19**			**Time to prepare together for a conversation (plan private environment; contact with risk manager; share views on event; plan what to say; anticipate response and need) **[69]	**Clinicians are better equipped for an effective conversation **[69]
**EAsf20**			**Training for clarification of difference between expressing regret and admitting liability **[50]**; of the pressures arising from instructions to give a partial apology (when staff would prefer to give a full apology **[54, 79]**); management of risks associated with tort system **[72, 81]	**Apologies are given with less fear/sense of risk **[50, 81] **of personal responsibility. Promotes that an apology is the right thing to offer regardless of review/investigation findings **[79]
**EAsf21**			**Knowledge of use of ‘appropriate words’ **[78] **/recognition of ‘profound effects of subtle changes in language’ **[75]** in disclosure meetings; use of established cognitive aid as best practice guidelines **[69]	**Clinicians will be better able to integrate own feelings into an honest account for the family **[78]** guidelines will improve (simulated) disclosure conversations, notably, posture/tone towards patient by experienced practitioners **[69] **Staff are more likely to have successful meeting **[58] **NB (Staff with best practice guidelines were more likely to apologise to patients [in simulations] however this training did not make the task of disclosure feel any easier for them **[78]**)**
EAsf22			Engagement of wider range of HCPs (e.g. for co-design of communication training) [75]	Different staff will realise that the challenges of disclosure work are common across health care teams (e.g. chaplains, clinicians, service managers) [75]
**EAsf23**		**Explanations (2 references)**	**Approaches that identify learning and ‘fair culture’(rather than apportion blame) **[49, 65]	**Staff will be less reluctant to report and disclose events **[49]**; the devastating effects of an incident that is hidden will be reduced; and opportunities for professional and service and personal learning are available **[49, 65]
**EAsf24**		**Navigation Strategies (3 references)**	**Named family contact/liaison has capacity (emotions and time) **[80]**; training and support **[52]**; sufficient influence and experience **[52]	**This contact will be able to work effectively **[52]**, responding to family needs throughout reviews/investigations (from routine updates to unmet expectations) **[80]
EAsf25			Clear pathways of contact/open communication with staff (raising concerns) developed by Trust [51]	Staff will be less fearful of contact with families with more compassionate communication and possibilities forcollaboration [51]
**EAsf26**	DISCLOSURE DURING REVIEWS AND INVESTIGATIONS	**When incident review and/or investigation initiated (11 references)**	**Standardised review tools and protocols that include communication with parents **[30, 34]**; dedicated support materials developed with parents **[30, 34]	**Staff will have guidance for when and how to involve a family **[30, 34] **NB (Staff feedback indicates more structured approach to review improves staff communication with parents **[30, 34]**)**
EAsf27			Chaired meetings with trained and experienced senior administratiors [71]	Meetings will be more reliable and robust [71]
**EAsf28**			**Dedicated/protected time for family involvement in reviews and investigations (and part of job plans) **[50]**; administrative support for reviews **[30, 34, 52]	**This work will be recognised as a necessary clinical responsibility **[50]**; with sufficient time, the quality of reviews will be improved **[50]**;** **less burdensome for investigators **[30]** (more time for discussion and identification of care improvements **[30]**)**
EAsf29			Professional duty of candour followed [50]	There will be more active participation in reviews (by staff as review leads and information-providers [50])
EAsf30			Systems that seek to reduce need for litigation against Trusts (e.g., early notification/compensation of costs) [81, 82]	There will be a reduction in fear of consequences of incident reporting and candour [81, 82]
**EAsf31**			**Training and expertise development for family involvement in investigations **[50]**; specialist training for investigators **[50, 74]** (national and mandated **[74]**); ongoing/facilitated team/peer-support programs **[74, 80]	**The competency of investigators will be improved **[74]**, including their confidence and resilience to effectively involve families **[50, 74, 80];**. These competencies of investigation and engagement skills **[74]
**EAsf32**			**Staff emotional support that is routinised **[61]**, dedicated, joined-up **[82]**, during incident investigation **[74]** and post-incident **[61, 74, 82] **Trusts (OH, Workforce Wellbeing and Board) responsible for provision of range of flexible care packages and specialist referrals **[82]	**Staff wellbeing will be better supported **[61]**; staff will be more likely to report and disclosure to a family next time **[82]**; trainee attrition might be reduced **[74] **NB (evidence of staff support offered in about 60% of NHS claims; no evidence of uptake or quality/continuity of support offered **[74]**)** **Support needs will be met as part of Trust-level duty of care to staff **[82]
**EAsf33**	OUTCOMES OF DISCLOSURE PROCESS	**Reporting and Feedback (2 references)**	**Informed of investigation progress and findings by key contact/liaison (not ‘kept in the dark’ **[52, 82]**)**	**Staff uncertainty and stress will be reduced **[52, 82]
**EAsf34**		**System-Wide Change/QI (3 references)**	**Evidence of corrective action/improvements from learning after incident (taken by teams/departments) **[50, 67]**; regular updates on shared lessons from reviews/investigations **[51]	**Leads to a reduction of stress in staff **[67]**; staff will feel that organisation is open with them; and they will be involved in learning for improvement **[51]
**EAsf35**		**Resolution of Staff (5 references)**	**Permission to communicate truthfully’ about event **[78]**; demonstrated effort by service to address harm to patient (amelioration) (taken by teams/departments) **[67]** with sincere apology and offer of compensation **[82]**; new systems for early notification/settlement of costs **[82]** dedicated and confidential post-incident support for staff **[49, 82]	**Leads to a reduction in staff stress, concern and trauma with the possibility of a just resolution **[67, 78]**; reduction of fear of litigation (‘barrier to safety’) **[82]**, anger is diffused and relationships with family might be preserved **[82]
**EAsf36**		Wider Revisions in Social and Healthcare Relationships	New practices (views on fallibility/expertise/care decisions) entailed in disclosure [8, 54, 63]	Will encourage new ways of working with staff and patients [8, 54, 63]
**EAsf37**			**Parents/families central in post-incident events and care **[76, 80]	**Will ‘upskill’ staff in new perspectives on user involvement in care planning **[76, 80]

**Table 5:** Explanatory accounts for improvements in open disclosure: what works, when and how from a service perspective (bolded explanatory accounts for services (eas) have been included in final c-m–o configurations (Table 6)

IDENTIFIED EXPLANATORY ACCOUNTS FRAM THE 38 DOCUMENTS				
**IMPROVEMENTS IN OPEN DISCLOSURE: WHAT WORKS, WHEN AND HOW FOR SERVICES**				
EXPLANATORY ACCOUNT FOR SERVICES (EAsv) REFERENCE	‘BEING OPEN’ PATHWAY	SITUATION	Indications of Mechanisms (forces, interactions, reasoning, and resources)	OUTCOMES for Services
EAsv85	EVENT IDENTIFICATION	Incident uncertain/unfolding	Routine invitation to discuss felt harm prior to discharge or during an assessment of reported symptoms [63]; standardised checks on women’s experiences embedded across maternity care pathways [63]; and family perspective included in clinical records and incident analysis [65]	Leads to the development of trauma-informed maternity service [63]; reduces the possibility of litigation by families who feel ignored [63] and the loss of vital information for patient care [65]
EAsv2			Extension of thresholds of harm (‘less’ serious incidents) [50, 63]; wider interest of improvement leads/committees in ‘trigger’ incidents [51] (with possibility of extension of these thresholds over time) [75]	Enhances view of service areas requiring improvement [50, 51, 63, 75]
**EAsv3**			**Following ‘Being Open’ guidance and Regulation 20 (Duty of Candour) **[32, 50]**with all reviews including a systematic and critical review of care **[58]	**Increases reporting of incidents **[50]**; improves discussions with families **[32]**; meets regulatory requirements **[50]**; creates more opportunities to learn from mistakes and substandard care **[50, 58]**and meets drive to improve maternity safety **[32]
EAsv4	ONGOING CARE AFTER EVENT	When the incident has happened and during ongoing maternity care	Organisation-wide [48, 78]staff training in Being Open purpose [48],policy/principles [49], and communication skills [78]	Leads to fewer possible repercussions for Trust (aggrieved families) [49]; workforce competencies are more widespread [78]; becomes more likely for disclosure to be enacted in local practice [48, 78]
EAsv5	DISCLOSURE PROCESS	Improvement Strategies and Infrastructures	Specialist, multi-disciplinary ‘event response team’ manage processes across service [49, 78] and immediate response to trigger events [78]; team selected by peers [78]	Disclosure processes will be more consistent/coordinated, there will be clear accountability [49, 78]; leadership positions/expertise will be developed [78]; a ‘tenants of disclosure model’ can be operationalised [78]; duplication likely to be reduced [49]and advice and standards more likely to be consistent [48]
**EAsv6**			**Concerted and resourced implementation strategy (including policy, guidelines, training, and evaluation of effect) **[48, 53]**, maximum use of IT **[77]**with whole service engagement **[75]**capacity to integrate patient experience intelligence **[51]	**Will meet the broad objectives of a pilot **[53]**; OD more likely embedded in organisation (not a discretionary activity) **[48, 75, 77]**; more effective identification of improvement focus possible **[51]
EAsv7			Comprehensive protocol/guidance (identification, disclosure, investigation, appropriate resolution) [78]	Meets one condition of programme implementation [78]
EAsv8			Dedicated, senior person to implement disclosure guidance (in Trust [51]; in regional partnerships [72])	Ensures clear and consistent leadership for implementation [51, 72]
**EAsv9**			**Gaining and sustaining senior medical ‘buy-in’ (with responsibilities for implementation and case reporting **[78]**) **[6, 9, 51]** and by local site engagement, with benefits evidenced to them** [78]** and local services having opportunities to adjust protocols to meet their own service conditions **[78]	**Encourages support by senior medical staff (required to promote uptake by colleagues **[51, 78]**; reassures junior staff **[6]**; is crucial to ongoing practice **[48]** and policy implementation **[78]
EAsv10			Disclosure identified as more than clinical competence and is identified as a service, organisational issue about workload, supervision, rapid organisational change [48], documentation [78], administration, and co-ordination [6]; there is communication/discussion and coordination of protocol and practice across units [78]	Embeds organisation-wide practice of openness [9, 78]; reduces burden of disclosure in individual clinicians; and enhances possibility of patient-centred disclosure practice [48]
**EAsv11**			**Trusts’ prompt referral of/comprehensive information on incident to external body **[32]	**Possibility of reduced litigation (parents get answers and/or assistance more quickly) **[32]
EAsv12			Organisational regulation [54, 80] with accommodation of differences in organisational maturity (how well systems support practice) [52]	Enables clear accountability for disclosure [80]; but variations across units are expected during early implementation [52]
EAsv13			Disclosure, apology, and early redress embedded in quality improvement work [82]	May reduce the need for the regulation of organisations [82]
**EAsv14**			**National frameworks/guidance on programmes for all Trusts and services **[51, 72]** (including for Board leads, staff skills, protected time, minimum data collection, and reporting requirements) **[51]	**Promotes a clear and consistent policy for family engagement and its requirements **[51, 72]**, combining specificity with flexibility **[72]
**EAsv15**			**Investments in staff education to address gap between disclosure guidelines and clinicians’ practice **[69]**, including supported space for clinicians and patients to negotiate the practical demands/contradictions of disclosure **[48]	**Effective disclosure becomes part of patient safety programmes **[69]**; and becomes more than ‘in principle’ agreement **[48]
EAsv16			Risk management formalised/embedded in improvement work/aspect of cultural change [49, 82] committed risk managers identified to embed disclosure protocol in each unit [78]	Incidents of disclosure are likely to increase [82]; evidence of impact of disclosure on reduction of incidents will be collected [49]; implementation of disclosure will be successful [78]
**EAsv17**			**Staff commitment to disclosure (notably, risk managers **[82]**, senior clinicians **[70]**, board and medical director/nominated consultant) with time and resources **[51]**; consistent communication of commitment **[78]	**Continuity of disclosure practice will be possible **[70, 78, 82]**; financial and HR investment in high-quality systems and processes more likely **[51]
EAsv18			Established provider service team reporting in Board and Commissioners into the divisions and ‘down’ to wards and local forums [49]	Develops high-quality safety assurance with grassroots identification of risk and improvement implementation [49]
EAsv19			‘Joined- up’ intelligence from reviews/incidents, patient experience, complains and support services by Trust Boards [50]	Enhances insights for safety improvement [50]
EAsv20			Adoption/development of legacy interventions (e.g., review tools, training, and engagement methods) [34, 51, 74, 77]	Creates a shorter/easier journey to improvement; interventions are more reliable [34, 51, 74, 77]
EAsv21		Ethos	Disclosure communication enacted as moral-ethical obligation of clinicians (not an administrative task) [54]; enacted in service-wide early response teams to encourage disclosure [78]	Embeds disclosure as an aspect of care s in each clinical service [54, 78]
EAsv22			Parents central in guidance [50, 74] and practice development [51]	Enhances effectiveness of guidance [50, 74]; strengthens partnerships with families [51, 74]
EAsv23			Change in NHS safety culture (with holistic work programme on structure, skills, capacity, and cultural reform) [52]; culture change in ‘healthcare micro-systems’ (over wider systems reform) [82]	Refocuses SI management from punitive/political process to learning for improvement [52] (52% of 2017 survey respondents said not yet achieved” [52]); different programs for Trust settlement after incidents possible [82]
EAsv24			Change in inspection and Board priorities from how investigations conducted and completed (within timeframe) to learning disseminated and embedded [50]	Practice will be valued for learning and improvement (not for meeting short targets) [50]
**EAsv25**			**‘High-level’ leadership in promoting ‘Just culture **[32, 50]**; desire to learn a central organisational value **[51]** (e.g. Provider Boards, Commissioners, and Regulators); embedded and consistent culture of openness/candour **[49, 58]	**Change more likely to happen within units **[32, 50]** when incidents, complaints, and concerns are seen as learning opportunities **[52, 88]** and when service-user experience is part of this learning **[58]
EAsv26		Organisation/Unit Legacies	When implementation approaches recognise the different capacities of organisations to drive attitude and practice change so that gradual and uneven change is expected in organisations [78] and varying degrees of foundational systems and expertise in organisations are anticipated [51, 52, 74]	Differentiated systems for support of staged implementation plans can be developed [52, 74]
EAsv27			Established success/experience in other family engagement practices [74]	Disclosure is more successful [74]
**EAsv28**		Governance	**Local Maternity Systems **[72]** and Health-Board/Trust buy-in **[75]** (with trained **[52]** executive and non-executive people leading these processes) **[62]**; resourcing is available **[77]**; there are clear and consistent guidance/standards/processes/tools **[50]** and time for development of expertise in their application **[74, 77]**; there is a Board-level family advocate **[51]** and minimum standard of training for all Board members **[52]	**Consistent disclosure improvements and learning are possible **[5, 50, 62, 77]**; investigating and learning emphasised **[52, 72]** in time (with variations between services expected) **[74]**; staff implementing family engagement are held to account **[51]
**EAsv29**			**Strong governance structures (e.g. review groups, including regular executive reviews **[51, 75]**; promotion of unit reporting for external benchmarking **[72]**; monitoring of training effectiveness **[72, 75] **and involvement guideline compliance **[72]**)**	**Essential for service improvement/learning and acting on lessons **[51]** and improvement monitoring **[72]
EAsv30			Commissioning that includes: lead for incident reporting and process improvement [50] and for maternity safety [32]; commissioners have time and training to quality assure disclosure and investigations [72]	More coordinated improvement work [50]; clarification of accountabilities [72]; family participation more likely to be achieved [72]
**EAsv31**			**Commissioners’ responsibility for investigation reporting/action plans with family involvement **[72]**; Board-level clarification and resourcing of Candour regulations (and inclusion of parents and staff in investigation processes) **[32]	**Regulation will be met **[32, 72]**; variability of investigations will be reduced **[72]
EAsv32			Inspection bodies include: mortality reviews/investigations [50]; compliance to family involvement guidelines [72] (e.g. to benchmark Trust leadership)	Improvements in national oversight and support for learning from failings; improvements in family involvement in national oversight would improve [50, 72]
EAsv33			Local Maternity Systems, supported by strategic partnership Boards, responsible for improving investigation process (and MVP involvement in it) [72]	National recommendations can be co-designed and included in local SI processes [72]
**EAsv34**			**Royal College clinical leadership and guidance to Trust/service investigators **[50]**; professional-led national quality improvement introduced **[62]	**Costs of external investigations teams (c£100 k per investigation) will be reduced **[50]**; national standards and objectives will be established **[62]
**EAsv35**			**Value of user-voice already established in organisation/clinical governance (co-production-user forums) **[51, 76]	**Reduction in the cultural resistance to involving families in making improvements in reviews/investigation processes **[51]** (however practice of user-involvement will always be more challenging than other aspects of clinical governance, especially where addresses difficult issue of ‘poor outcomes’) **[76]
EAsv36			Networked governance structures to enhance disclosure practices (e.g. Board-level, Membership Councils, QI Steering Groups; Patient Leads) [49, 72]; annual reporting of national bodies to include lay summaries [62]	More effective learning and engagement for Sis and involvement of families [49, 62, 72]
**EAsv37**		**Accessibility/Availability**	**Family-centred approach to engagement in reviews and investigations **[50]**, including information materials noting multiple opportunities to engage **[34]**; and staff training in this perspective **[76]	**Increases satisfaction of families **[50]**; family engagement is improved **[34]**, care planning and delivery are improved **[76]
EAsv38			Culture that supports meaningful apology for any harm [49, 50, 81] and explanation of circumstances without blame [81], including legal protection [82]	Reduces likelihood of escalation or legal claim [49, 50, 81] NB: (limited potential to reduce malpractice claims by US families with birth-injured infants) [82]
EAsv39		Explanations	Comprehensive assessments of care during review [72]; correspondence in care standard assessments (between services and external bodies) [32]	Delays in settlements for families are mitigated [32] (possible reduction of costs) [32]; learning from cases for care systems improvements are increased [32, 72]
**EAsv40**			**Inclusion of family and carer understandings of events **[50, 80]**, with understanding that common understanding of what happened might not be reached **[80]	**Increases opportunities for learning from family experience of care across complete care pathway) **[50, 80]**; reduces possibility of ongoing conflict if family listened to **[80]
EAsv41			Investigations include clinical and legal experts (examining all relevant documents) [32]	Investigations can bridge ‘claims, safety and learning functions of the organisation’ [32]
**EAsv42**		Consistency in Disclosure Process	**Formal, family engagement guidance (shared between services and between external organisations) **[32, 80]**, and review tools **[62]**, are co-developed with staff and parent advisors **[34, 77]	**Leads to more consistent information and shared resources **[80]** that are relavent **[34, 62, 77]**, avoid duplication **[32]**, and are available to the service**
**EAsv43**		**Navigation Strategies**	**Named professional/patient representative or advocate to manage co-ordination of information between parents and clinicians **[71, 75]	**Leads to the provision of crucial infrastructure for improvement of ‘Being Open’ guidance **[32]** (more information and relational consistency between Trusts and family **[71]**)** **NB: (unclear if that person should be ‘fully independent’ of clinical team) **[71, 75]
EAsv44	DISCLOSURE DURING REVIEWS AND INVESTIGATIONS	When incident review and/or investigation initiated	Investigation Leadership that is expert in family liaison and includes risk management /governance team (not consultant in charge) [34, 49]	Enhances the reliability and consistency of findings [49]; the incorporation of action plans into clinical governance plans [34] and findings more likely to be underpinned by ethos of candour [51]
**EAsv45**			**Robust review/investigation process including whole care pathway (multi-agency **[59]**; cross-department **[34]**; multi-discipline **[34, 62, 74, 76, 79]**); parents’ perspective **[34]**; external or independent peer-review **[34, 72]**, and adequate RCA methodology **[72]	**Enhances learning from the incident by more comprehensive for improvement planning **[34, 59, 62, 74]**; encourages care variation and grading from a multi-disciplinary perspective **[34, 79]**, along with the use of ‘fresh eyes’ to identify systems issues **[34, 62, 72]** to identify active and latent failure **[72]** and the wider development of cross-sector relationships **[76] **NB (but 17% reported PMRs 2018–19 completed by 1–2 same discipline clinicians **[34]**; 1:5 PMRs 2018–19 had external member input **[34]**)**
EAsv46			Planning [50] and training [79] for multi-disciplinary/sector review/investigation (establishing ToR, leadership, expectations of contributions and time-lines reflecting complexity [34, 50], and building of cross-sector relationships) [50]; investigators trained in RCA techniques [50]	Enhances reliability of review/investigation processes and completion in a realistic timeframe [34, 50, 79]
EAsv47			Independent, structured peer-reviews underpinned by just culture approach [72]	Reduces risk of ‘political highjack’; increases possibilities for the identification of systems-factors in development of action plans [72] NB (costs estimated as £2,100 per peer-reviewed case) [72]
**EAsv48**	**OUTCOMES OF DISCLOSURE PROCESS**	**System-Wide/QI Resolution**	**Board and trusts governance teams invested in action planning for post-review ongoing quality and safety improvement **[34]	**Shared ownership of actions and system-level changes more likely **[34]
EAsv49			Focus of national bodies on improvement processes rather than completion deadlines [50]	Reduces focus by Boards on more immediate targets and greater focus on longer-term systematic change [50]
EAsv50			Integration/standardisation [50] of (internal; external) data collection/surveillance systems [53]; robust mechanisms to disseminate learning from investigations or benchmarking beyond single Trust [50] (e.g. across local maternity system); beyond single external bodies [32]; administrative support for Trusts to engage [32]	Increases opportunities for national learning from local reporting [53]; possible reduction in repeated mistakes [50]; more rapid learning [32]; engagement possible [32]
EAsv51			Ongoing review process/audit spirals or cycles [62]	Supports (re)evaluation of recommendations and their implementation [62]
EAsv52		In-Case Resolution	Meeting ongoing care requirements [80], including offer of fair compensation, and if admission of fault [82], costs payments [74, 81], and informed sign-posting for expert follow-up [80]	Diffuses anger towards individuals or service and may help to preserve relationship with family [74, 80–82]
**EAsv53**			**Trust/employer recognition of duty of care to affected staff **[32]**; investment in dedicated joined-up post-incident support **[32]**; changed perspectives staff HR during investigation (e.g. time off work not a penalty) **[52]	**Leads to the development of joined-up and dedicated systems for effective post-incident staff support /workforce wellbeing/OD improvement **[32]**; staff less traumatised/likely to feel penalised **[52]**; staff more likely to be retained **[32]
EAsv54		Wider Social Influences	Professional insurance policies support participation in disclosure procedures [78]	Impact/use of disclosure protocols increases; organisations promotion of disclosure work and systems/team perspectives on issues for improvement not undermined [78]
EAsv55			Litigation fear and costs managed [72, 78] (e.g. protected spaces [50]); external agency interventions [32, 82]	More reviews happen [72]; open communication is more likely (expected to reduce complaint and litigation need [32, 50, 82]; evidence that decreases malpractice costs [78]; legal duty not breached [50]
EAsv56			Consumer-perspective on incidents (personal/psychological [63]), disclosure, involvement routinised [54]	Consumer experience is incorporated into wider patient safety issues [54]; ‘cultural shift’ from bio-medical perspectives on incident [54, 63]
EAsv57			Increasing public pressure on policy makers [53]; costs of clinical negligence claims (connected to marginalisation of families) [32]	High-level drivers on organisations to secure disclosure improvements [32, 53]

Table 6 presents the results of the five stages of data analysis and synthesis, including the consolidation of the 68 coded EAs to mechanisms and their various relationships to context and outcomes. Further details of these mechanism sets in realtion to context and outcomes is presented in Table 6.

**Table 6:** Five programme theories for improvements in open disclosure.*(c-m–o* configurations identified from eas relevant to families (eafam, see Table 3), staff (easf, see Table 4), and services (eav, see Table 5)

INITIAL PROGRAMME THEORY	Context		Mechanism		Outcome	
	*Incident*	*Institutional Conditions and Systems*	*Resources, constraints, and opportunities shaping this element*	*Reasons, responses, and assumptions involved in this element*	*More immediate changes in experience, perspectives, and behaviours*	*Longer-terms changes in perspectives, values, and practices*
**MEANINGFUL ACKNOWLEDGEMENT THAT HARM HAS HAPPENED**	Circumstances and conditions of harm identified (irrespective of whether this is avoidable)		-Senior leadership buy-in to implementation of OD (EAv6)			-OD becomes embedded as a taken-for-granted aspect of clinical care (EAv6)
		Legislation for disclosure of some incidents (EAv3) Professional Duties and Codes of Conduct for disclosure (EAfam2) Incentivised safety improvement schemes with prescribed thresholds for disclosure (EAfam2)	-Expert clinician availability, time, attention, and continuity for initial and subsequent family meetings (including meeting preparation time) (EAsf31;5) (EAv6) - ‘Safe space’ for the lead clinician to undertake a formal meeting with parents, without fear of litigation (EAfam18, EAsf11)	-Honest, timely, and personalised acknowledgment of harm to the family that includes empathic apology in context with an ongoing clinical relationship; sensitivity to the family’s needs for further discussion and recognition of/meeting family entitlement to NHS compensation (EAfam1;2;18;33) (EAsf21) -Involvement of family in disclosure conversations and processes organised around their situation and needs (EAsf31)	**For families…** -Might recover family trust or confidence in the clinician or the service (EAfam2;18) -Reduces secondary harm (by improved incidence of disclosure) (EAfam1;33) (EAv3;6) -Families are less likely to always feel aggrieved (EAsv3) **For staff…** ***-***The trauma and anxiety of the event may be alleviated if an incident is discussed openly with a family (EAsf18)	**For families…** -Reduces damage to wider health care relationships caused by not recognising/ignoring harm done (respect for family experience) (EAfam2;18) May lead to active and more satisfying participation in reviews/investigations and inclusion of incidents defined by family as significant (EAfam2)
			-Service investments for developing and sustaining expertise and confidence of clinicians engaging with injured families (EAsf10;18) (EAv5)		**For families…** -Possibilities for more families to have a voice in disclosure conversations, to attend meetings, and to be heard (EAsf10) **For staff…** -Increased confidence and expertise in undertaking disclosure (EAsf10)	**For staff…** -Increased confidence and expertise in undertaking other sensitive meetings and conversations (EAv5) -May result in greater awareness of family-defined events of harm and care (EAfam6) (EAsf5)
			-Inter-professional, intra-service and inter-service working to recognise emergency of harm over time (EAv45) -Pre-discharge assessment of possible harm to a family (EAfam6)		**For families…** -Possibilities for the family to identify and report harm and receive a sincere and relevant response to their concerns in situations that would ordinarily be unknown to the service (EAfam6) (EAsf5) -May lead to more timely reparations (treatment or compensation) (EAfam2;6)	**For services…** -Increased service investments in guidance and staff (EAv9) -Creates possibilities for service learning (EAv3;45) -OD becomes embedded as ongoing and wide-spread clinical activity (EAv6)
**CLINICIANS WHO ARE SKILLED IN OD**		Professional and organisational obligations to conduct empathic disclosure with families (EAsf8;20) -Employer obligations to staff (EAsf20)	-Service investments in specialist communication training and its commissioning for clinical leads (EAfam17;18) (EAsf9;31) (EAv15;17) -Cognitive aids to support disclosure conversation (EAsf21) -Time to prepare for disclosure conversations with families (EAsf17;19) Ongoing peer support (formalised in mentorship) for OD practice development (EAsf31) -Availability of time during staff induction and in-service meetings to disseminate best practice examples of disclosure with families (EAsf5) -Coordinated investment in learning between clinical and corporate leads to carry an ‘organisational ethos’ of no-blame (EAsf1;4;12;23)	-Staff who are committed to OD as a practice (EAv 15) and who are able to ‘bridge the gap’ between in principle agreement and practice change (EAv17) (EAsf14) -Learning by mentorship and role modelling (EAsf13) -Confidence to innovate aids and guidance in response to events and family situations (EAsf26) -Staff have the opportunity and authority to disseminate new approaches to wider clinical team (necessary for revisions of practice) (EAsf35) -Availability of clinical leads to mentor junior staff in disclosure skills (EAsf7) (EAv10)	**For families…** -Expertise and felt safety of clinicians is necessary for meaningful apology and open conversation, which impacts families (EAfam17;18) -May mean that a family feels recognised when guidelines improvised to their needs (EAsf26) **For staff…** -Anxiety and uncertainty (emotional toll) around encounters with harmed families may be reduced; more positive relationships with families may be possible (EAsf13) -Increases confidence and competence in disclosure conversations; relationships with family may be preserved (EAsf13;17;19;31)	**For families…** -Possibility of more widespread openness in senior clinician responses to events of harm and enquiries about harm (EAsf14) **For staff…** -Could encourage staff to trigger formal response of suspected adverse event (EAsf1;32) -Openness to families more likely to become the ‘mind set’ of practitioners (EAsf13) -Emotional and social support needs (during investigation and post-incident) will be met by teams on a routine basis (EAsf4;6;20;33)
**CLINICIANS WHO FEEL SAFE PRACTICING OD**			-Post-incident clinician support to explain events (individual and team debriefings) (EAsf33) -Availability of joined-up and consistent post-incident emotional support during incident investigations (including commissioning of appropriate post-incident care/counselling support if required)(EAsf6) (EAv53) -Dedicated post-incident support for individuals (educational supervisors or commissioned services) (EAsf32); organisations meet duty of care to staff (EAv53)	-Trust in colleagues, managers, and educators to seek emotional support during investigation (EAsf32)	**For families…** More likely that disclosure will happen in the future (EAsf33)	**For staff…** -Possibility of revised perspectives on infallibility (and recognition of clinician needs for emotional care) (EAsf36) **For services…** -More likely to retain trainees and staff; more likely to embed meaningful disclosure practices as ‘taken-for granted’ aspect of patient care (EAsf5;32) (EAv10;53) -Desired practice of OD more likely to be supported by staff (EAv10)
**FAMILY INVOLVEMENT IN REVIEWS AND INVESTIGATIONS**		National and local programmes for examining events of harm that seek to include family questions or perspectives during incident review or investigation processes (EAfam7) (EAsf31)	-Organisational governance and professional leadership promoting family involvement in the process (including the family voice) (EAfam9) (EAv14;25;28;29;31;32;34) -Dedicated time for named clinician or independent person to act as an advisory ‘link’ between family and organisations (EAfam23;24) (EAsf24;28) (EAv43) -Family-centred/open-door policies for involvement (EAfam12;32) -Guidelines for staff for family engagement processes and use of these guidelines (EAfam7;34) (EAv42) -Family advocacy (service or charity based) representing family concerns (EAfam31) (EAv35;42) Family navigator systems (EAfam23;24) -Spaces for cross-service working (e.g., with GPs and bereavement specialists) to address longer-term family needs (questions and conversations) (EAfam28) -Provision of interpreters (EAfam3;7) Availability of family therapeutic support during process (EAfam31) Family-centred care pathway with post-discharge care planning (EAfam28)	-Service ethos of family involvement promoted in governance and to professional staff (EAfam8) -Personalised approach that increases availability and accessibility of involvement in review/investigations is possible (EAfam12); disclosure processes are explained in a way the family understands (EAfam13), face-to-face, with time and space for the family (EAfam15;23;24); continuity of family involvement (EAfam23) -Relational care of the family (responsive to situation, background, changing needs, circumstances) (EAfam12;15;17;23;24) (EAsf4)	**-For families…** Do not have to ‘chase’ information on their review/investigation (EAfam8) and mistrust is reduced (EAfam7) -Clarification of processes reduces confusion and mistrust (EAv42;34); gives the family opportunity for questions (EAfam14;15;23;24;26;28) -Engagement in process can be adjusted to the family’s needs, interests, and situation (EAfam12;15;32) Specialist support for social diversity and/or emotional needs necessary for some families to be involved in investigation process is provided (EAfam7;12;31) -Family perspectives and questions represented during reviews and investigations (EAv40) **For staff…** -Clarification of processes (EAv42) -Family involvement is an aspect of the clinical or independent role (not discretionary) (EAsf28) **For services…** -May prevent complaint or litigation by diffusing anger, but may increase demands on the service by an expert family advocate (EAfam24)	**For families…** -May strengthen consistency of routine practices of family involvement throughout reviews and investigations, including family-centred approaches to this involvement (EAfam9) (EAsf24) -Possibilities of active partnership working with clinicians and/or services (EAfam8) **For staff…** -May increase knowledge and confidence and decrease the emotional demands of working with harmed families (EAsf28) -May enhance interest and commitment of staff to involve families in reviews/investigations (EAv14;25;35;40;42) -May establish new perspectives on family/staff relationships (EAsf28) **For services…** -May strengthen family-centred approaches across the service more generally (EAv28;40)
**MAKING SENSE OF WHAT HAPPENED**		National and local programmes for examining events of harm that seek to include family questions or perspectives during incident review or investigation processes (EAfam7) (EAsf31) Availability of different frameworks for incident reviews and investigations in maternity care (EAfam22)	-Policies and guidance for incident review and investigation reporting, that include family perspective (EAv45) -Protocols and incentives for and prompt referral to other reviews and independent investigators (EAv11) -Organisational support and resourcing for clinicians to respond to family questions about investigation findings (EAsf7;17)	-Incorporation of family perspectives on the event (these may differ from service perspective or clinical records (EAsf17;19) (EAv14) or may be used to supplement these views and records) (EAfam26) (EAsf10;36) -Multiple explanations may be presented to family and explained in the integrated report (EAfam20;22) -May align the expectations of families with what is possible (EAfam19) May establish some negotiated understanding with families who require individual accountability for the incident (EAfam19) (EAsf10)	**For families…** -Report is accurate and accommodates family perspective, is complete and free from jargon, and is forwarded to families before being forwarded to organisation (EAfam35) -When shared understanding can be agreed, a sense of resolution, relief, lifting of guilt, and less mistrust of clinicians or service is possible. When disagreements over events continue, then further distrust in clinicians or service results (EAfam19;20;22) (EAv11;45) **For staff…** -Increased confidence to discuss the event with colleagues; where a ‘fair culture’ approach is taken, staff feel less fearful of blame or loss of reputation (EAsf10;17;19) -When family expertise is incorporated into the understanding of the event, skills in responding to family perspectives and concerns are enhanced (EAsf26) **For services**… -Development of different or more comprehensive understanding of an event because of the family’s contribution (EAv45) -Possibility of reduced litigation when families have answers to their questions and prompt referrals are done (EAv11)	**For families…** -Families more likely to feel confident in the process and in the honesty of the service (EAv14) -Greater possibility for securing practice and service improvement that includes family experience (EAfam22) Revisions in public understanding of clinical authority and infallibility (EAfam20) **For staff…** -Revised perspectives on bio-medical authority and infallibility (EAsf36) -Open discussion of adverse incidents is normalised (without immediate fears to reputational damage) (EAsf7) **For services…** -Family expertise and experience possibly available as additional learning resource (EAfam26) If disagreements continue, then possible reduction of distrust and legal action by families (Eafam19; 22)
**SEEING THAT THINGS HAVE CHANGED**			-Comprehensive and structured organisational investments in OD (candour training, guidelines, leadership) to enhance openness for the purpose of systems-improvement (EAv45) -Increased organisational accountability for acting on systems-errors (external monitoring and benchmarking) (EAv12)	-Embedded open (not defensive) responses to families and to incident reporting for service improvement (EAsf35) -May encourage or undermine openness to families and organisations depending on implementation (EAsv35)	**For families**… -Reassurance that lessons have been learned; might help to make sense of loss (EAfam36); clarification of service accountability (EAv11;EAv48) **For staff**… -Reported reduction in post-event trauma when corrective actions after the incident are taken and evident (EAfam36;9) (EAsf35) (EAv11) **For services…** Enhanced learning (EAv45;11;12)	**For families…** -Organisational commitment to (or demonstration of) change because of systems failings might generate new perspectives on user involvement in education and services (EAfam9) **For staff…** -Ongoing normalisation of discussions about incidents (between colleagues and with families) might be possible (EAsf1;7;23) -Families involved in updates on post-incident actions/accountabilities (EAfam36)
			-Revision of management, corporate, and inspection priorities from completion of narrow deadlines to demonstration of learning towards systematic improvement (EAv48) -Development of senior risk and safety teams (aligned with quality improvement teams and governance, including family representation) (EAfam9) (EAsf1;7) (EAv48) -Protocols and incentives for (and prompt referral to) other reviews and independent investigators (EAv11)	-Shift from improvement as a short-term target/completion deadline to an ongoing process (EAv48)		**For services…** -Safety challenges and recommended improvement strategies are more visible and the is an investment in their long-term completion (EAv48) -New practices of service accountability to the public, including harmed families, might be developed (through user-voice in Board, Council, and QI meetings and collaborative improvement work) (EAfam9) (EAv50)
			-Cross-boundary/whole pathway working (e.g. primary care and counselling, for communication and learning about incidents in maternity units (EAv50;EAfam28)	-Requires cross-sector clarification of leadership, investigation methodologies, and approaches to learning and accountability (EAv50)	**For families…** Recommendations more likely to reflect their experience of an incident (EAfam28)	**For services…** *Ongoing opportunities to identify more immediate and longer-term practice and service failings (EAv45;50)
			-Individual patients or families with opportunity and networks to press for change in a unit or across the wider service to address events like those they experienced (EAfam9)	-Family with social capital to influence professional leads and with motives and networks for dissemination of learning (EAfam28)	**For families…** -Learning alleviates the harm of the incident (EAfam28) -Recognition of some individuals’ or families’ ‘expertise by experience’ by professional bodies and their members (EAsf23) **For staff…** Learning from incidents (EAfam28)	**For families, staff and services**… -Safety improvement might happen in some services (EAfam28) -Development of staff skills and awareness of the value of family insights, including informing safety and care priorities (EAfam28)

### Narrative summary of contexts and mechanisms for strengthening OD

Our analysis identified three contexts that influenced the triggering and outcomes of the key mechanisms identitied. These were: (a) the configuration of an incident (how and when it was identified and issues of severity); (b) national or state drivers, such as polices, regulations, and schemes designed to promote OD; and (c) the organisational context in which these drivers are recieived and negotiated. Given the focus of the synthesis we agreed with our stakeholder groups, national interventions comprised the context rather than the mechanisms for impovements in local OD practices.

### Programme theories

The following sections describe each of the five mechanism sets, in relation to these three contexts and as an initial programme theory.

#### Receiving a meaningful acknowledgement that harm has happened

##### Initial programme theory

When a family feels that their experience of harm and its aftermath has been acknowledged in a meaningful way, their trust in their clinicians and the service is more likely to be rebuilt. In addition, clinicians feel less anxious about the event and about their relationship with that family.

Regardless of the circumstances of harm and the organisation of services, the early and meaningful acknowledgement of harm was a critical aspect of OD identified in EAs for families (*n* = 5); staff (*n* = 7) and services (*n* = 4). Meaningful acknowledgement was emphasised as including recognition of the uniqueness of the experience and its aftermath on a family. This expectation of meaningful acknowledgement of harm involved clinicians recognising and understanding the experience of the family and was additional to the professional and regulatory duties of apology concerning clinically defined incident thresholds [32, 50]. The rationale for this acknowledgement differed from the organisationally and professionally prescribed OD tasks of giving honest information and explanation of what happened and from family involvement guidance, in which the clinician’s primary responsibility is to ensure that the family is invited to ask questions or raise concerns [34, 75]. Three EAs stressed the importance of a family-centred perspective on the severity of harm and its aftermath. Only one paper considered the possibility that injured families may introduce clinicians to alternative perspectives on harm during their involvement with services [48].

As part of the meaningful acknowledgement of harm, the value of an honest and direct apology to a family during initial and subsequent OD conversations was noted in EAs relevant to staff and to families extracted from six papers [51, 54, 62, 65, 67, 75]. Sometimes a sincere expression of regret was found to enable some restoration of trust in a clinician or the service for the family [54, 65]. Indeed, clinicians expressed surprise and relief that a family might sometimes offer understanding after an honest expression of regret [6, 75]. Several studies indicated the disappointment of families when these apologies did not translate to their subsequent experiences of care. It was reported that many families felt the injustice of poor ongoing care and expressed that they felt insensitivity from general healthcare staff to their trauma and loss [61, 83].

When evidence of harm was clinically uncertain (for example, in some events of birth asphyxia of babies) and so evidence of harm and extent of harm was established over time, meaningful acknowledgement by a clinician was more complex and sometimes involved expert diagnosis and discussion with families and a wider clinical team [58, 62, 64]. Additionally, maternal harm or significant harm to babies was sometimes identified weeks or months after the incident. This meant that OD conversations must be initiated by clinicians or services far removed from the originating events and the clinicians involved [32, 63, 65]. These aspects of ongoing, multi-professional, multi-service OD work raise challenges around trust and communication with affected families [65]. Interventions that aided recognition by staff were appreciated. Post-delivery assessment, along with cross-service co-ordination and cross-unit collaboration, were important for harm to be identified and disclosed by appropriate staff and services over time. At the same time, regulatory or procedural edicts could determine different clinical types or levels of incident severity that required OD. For example, in England, healthcare organisations carry no legal obligation to disclose incidents to a family when these incidents are not classified as causing moderate or severe clinical harm [23]. The identification of an incident over time and co-ordination of OD requires clinical information, time, and collaboration with a family to understand and discuss events that are hidden or less immediately obvious. Three studies explored the experiences of families after stillbirth, noting experiences of marginalisation, unrecognised distress, and the ignoring of their distinctive needs [61, 62, 64].

Two papers reporting results from the same study found that the timing and conduct of OD meetings with families were often indicators to those affected of how seriously the event and its impact were taken by that service [48, 54]. Creating the space and time for exploration and discussion of events and their consequences communicated acknowledgement of the family’s situation [48]. Family preferences for the presence of certain clinicians at their OD meeting also suggested the importance of personalising these events from the perspective of the family. While families more often want to meet with a senior clinician already known to them [64, 73], some also want to meet those directly involved in the incident so that they better understand events and their aftermath [54, 69] or can receive a more personal expression of regret [54]. A recognised barrier to meaningful acknowledgement during OD meetings was the inhibiting effects of clinicians’ worries about the risk of disciplinary action or litigation following OD conversations. The distorting effects on conversations where legal or organisational representatives were present, or where legally protected ‘safe spaces’ were uncertain, limited the possibility for openness and honesty [50, 81].

The meaningful acknowledgement of harm was secured by the conversational skills of empathic clinicians in cases where families might accept an honest expression of regret and explanation of what happened [54, 65]. However, when a family needed material compensation or assistance, uncomplicated and timely settlements by the service were also important for diffusing anger and the chances of litigation, as well as for preserving clinical relationships [32, 81, 82]. More immediate, short-term assistance with ‘out-of-pocket’ expenses, along with the provision of any further or specialist care, were valued as expressions of acknowledgement of harm [54, 74, 80, 81]. Surprisingly, few included papers considered the divisive effects of adversarial investigation and litigation processes on clinician-family relationships after harm in maternity care. Yet, these could shape ongoing suspicion between families, clinicians, and services, especially when it was felt that a genuine acknowledgement of harm did not take place after an incident [32, 81, 82].

#### Family involvement throughout reviews and investigations

##### Initial programme theory

When families have a representative, if they choose, to help them navigate review and investigation processes, they are less likely to feel alienated and distrustful of services and are more likely to be heard in discussions about the event and their care.

Eight EAs (for families *n* = 3; for staff *n* = 2; and for services *n* = 3), identified from 10 documents [6, 32, 50, 51, 62, 73, 75, 77, 80, 89], highlighted the value of a named, expert, family contact to act as the ‘link person’ through organisational processes, individualised care, and information-giving. The importance of personalised and ongoing care was identified within the overall context of wider national and local programmes that sought to involve families in review and incident investigation processes. Family navigator systems, family advocacy schemes (within or beyond health services), and the resourcing of cross-service working opportunities and of open-door policies for families were additional structured approaches to family involvement [50, 80]. Resourcing of assistance to families for their involvement, for example the provision of therapeutic support or language interpreters, was not extensively noted. The named family involvement role kept families present and visible within busy services [79], where unexpected delays and complications in bureaucratic processes might not otherwise be explained to them [71, 81], and could cause further upset and suspicion [50, 82]. However, this role was expected to do more than keep a family up to date with the process of their case. In this dedicated role, liaison personnel responded to the particular and changing situations and needs of a family and represented family interests and perspectives during review and investigation meetings. Furthermore, it was anticipated that a service ethos and situations for relational care would enhance the inclusion of family perspectives and questions in reviews or investigation, so that active partnership working between clinicians and families could become possible. One study [23] acknowledged a general point that the invitation to a family to raise questions about what happened, does not, in itself, ensure meaningful or empathic family involvement [50].

Although the value of a named support person for families was frequently suggested as an important element of OD, the composition, boundaries, and implications of this role as an advisor, information-giver, or family advocate were not fully explored. The requirements of this role were only briefly noted as ‘training and support’ [52, 77] and protected time [80]. The legal implications of family advocacy were not explored. The anticipated duration of family involvement with a service after an incident, along with the duration of a ‘named link’ relationship with a family varied considerably in the literature. For example, in the case of a neonatal or maternal death, some suggested closure at discharge from a service [50, 80], and others proposed that the relationship be sustained until inquest or retriggered on future readmissions to a service [50, 71, 73, 80]. Inherent tensions between the responsibilities of the ‘named link’ were rarely discussed in the identified papers. For example, the work of the named link might span from care coordination to family advocacy, with different implications for families depending on the context. Some review and service redesigns identified bereavement midwives [73] or community midwives [75, 77] as the named links for families because of their ability to champion or translate the concerns or questions of a family to the clinical teams more effectively than non-clinicians [71, 75]. However, the expectations of the named link’s employers, managers, peers, and wider professional assumptions and identities may be in direct conflict with their role as family advocates. The development of the role of a fully independent family advocate is not fully explored or evaluated in the identified literature, but it is noted as a possibility for families in better-resourced maternity units [71].

The wider significance of keeping affected families informed and updated on review and investigation processes was widely discussed. These studies focused less on issues of family entitlement to knowledge and understanding and more on the challenges of producing and circulating accessible, written, standardised guidance to families with differing needs and expectations [30, 31, 58, 59, 71, 73]. This guidance highlighted the shortcomings of some services that neglect to provide family-centred advice [31, 32]. Information content and delivery, designed with staff and parent advisors, was expected to have greater relevance and desirability for families [29, 34, 77]. However, prescriptive, standard information for families about review and investigation processes was often considered inadequate. For example, guidance for families on recommended time-frames for review/investigation completion could be reassuring to families but was also found to enhance disappointment and distrust when delays happened [50, 82]. Furthermore, in some circumstances, families felt irritated or confused when information was duplicated or reinforced multiple times by services [32], however in other cases, this duplication was necessary for families in shock and crisis who did not grasp information the first time it was shared [34]. These findings suggest the importance of personalised information sharing rather than standardisation.

Similarly, the adaptation of guidance literature in response to social diversity [59, 76], including the provision of translation [80], was seen to ‘solve’ the task of recognising family differences [50, 80]. However, others found that this approach may overlook more fundamental concerns about family expectations of OD in relation to socio-religious background [60]. Four studies made clear that for pre-designed information materials for families to have relevance and resonance, they had to be introduced and discussed during ongoing OD meetings, ideally by a clinician or advocate who already knows that family [54, 59, 60, 73]. One paper identified the need for the development of a family-centred pathway for embedding pre-discharge routines of post-incident enquiry and care planning discussion in maternity services [63].

#### Making sense of what happened

##### Initial programme theory

When families feel that they can make sense of what happened and that clinicians and services have also sought to do this, they feel less dismissed; both they and others affected are more able to begin some recovery.

Fifteen EAs, identified from 15 documents, highlighted that a crucial and ongoing aspect of OD was addressing families’ needs to understand the events that happened to them [30–32, 34, 49, 50, 58, 59, 62, 64, 65, 70, 71, 73, 80]. As described most frequently in the case of baby loss, most families also sought to make sense of a ‘life shaping’ event in ways that extended beyond the services where incidents happened. However, the explanations offered by services could reduce family distress and mistrust in health care, help some families to recover from grief [62], and begin to plan for the future [60, 62]. However, not uncommonly, families felt that explanations given were incomplete, misleading, or incompatible with their understanding of what happened [6]. As described most frequently in the case of baby loss, most families sought to make sense of a ‘life shaping’ event in ways that extended beyond the services where incidents happened [64]. Not all reviews or investigations could establish causality [58] or had sufficient scope to address all questions raised by a family [71, 80]. Systems-based explanations of what went wrong could disappoint families, who felt that personal behaviours were most important [80]. When incidents were reviewed or investigated using different approaches, there could be inconsistent views on how causality was explained. This difficulty was addressed in several EAs. One identified the importance of clarification to families of all investigation routes and their organisational hierarchies, so that complexity or contradiction was reduced [52]. Another argued the need for ‘expectation management’ of families, so that they were informed of the limitations of the incident investigation [80]. Another advocated for the future production of single, integrated reports that would reduce family experiences of discordant interpretations [34]. These differing approaches indicated wider assumptions about families as recipients and contributors to understanding incidents. One paper identified the potential significance of clinicians’ reflective inclusion of harmed families’ experiences and expectations of incident reviews to encourage wider re-thinking of the relationship between clinical authority and family experience and expertise [6].

##### Initial programme theory

When clinicians are skilled and feel safe to conduct disclosure conversations with families, such conversations are less likely to be avoided and are more likely to become embedded in ongoing clinical practice, and issues of responsibility are more likely to be addressed.

The specialist communication training for senior clinicians conducting OD with families was identified as an important resource in 14 EAs (with identified outcomes for families *n* = 3; for staff themselves *n* = 9; and for services *n* = 2), extracted from 16 documents[31, 32, 48–50, 52–54, 58, 65–69, 75, 78]. Embodied communication skills, including active listening, the language chosen, posture, and conversational tone were noted as crucial for initial and ongoing interactions with injured families [32, 54, 58, 65, 66, 68, 69, 75]. The required expertise to anticipate and improvise these conversations was also noted in these papers. While ‘best practice’ communication guides and protocols were described as important resources for both senior and junior clinicians [66, 68, 69], the wider context of variability of events, including family circumstance, was also noted as an aspect of situated clinical judgement [48]. Improvisational skills were crucial for OD to become more than an ‘in principle’ agreement and to be enacted in differing event and organisational contexts. OD communication training for clinical trainees, for labour and delivery clinical teams, and multi-disciplinary OD leads was shown to increase self-reported confidence, competence, and cross-disciplinary collaboration in conducting initial and ongoing OD conversations. An EA in one paper [68] posited the connection between these effects of training and a reduced risk of workplace burnout for clinicians. One study found that while training clinicians to use ‘appropriate words’ did not make the task of OD feel easier, it helped them to express their feelings in ways that encouraged a more honest conversation with families [78]. This could indicate that the performative skills and personal and moral aspects of OD conversations both require careful nurturing.

Three EAs identified mentorship, with time and space for the dissemination of best practice examples of OD, and role modelling as important resources for embedding openness with families in team and unit practices. Skills and awareness training across clinical teams, beyond training dedicated OD leads, was also identified as important for openness to families to become part of the ‘mind set’ of practitioners [53, 78]. More generally, one EA, identified in a systematic review, suggested that the inclusion of more junior or non-specialist clinicians in incident review meetings was connected to the demystification of OD and investigations, and could potentially alleviate fear that they would be blamed by their colleagues or families when incidents occur [67].

Twelve EAs identified that post-incident support for clinicians could improve outcomes for families (*n* = 1); staff (*n* = 8); or services (*n* = 3). Post-incident needs ranged from the inclusion of staff in updates on the progress and outcomes of reviews/investigations affecting them to updating them on team or departmental changes resulting from review and incident investigation reports. Clinicians’ knowledge that changes would be made was associated with a reduction in their post-incident trauma. One EA, identified in a systematic review [67] proposed that the exclusion of affected front-line staff from OD and investigation processes may heighten post-event anxiety, fearfulness, and felt isolation. A related EA in four documents posited the relationship between staff experience of no-blame processes and their lessened worry and uneasiness when disclosing, as well as reporting, future incidents [49, 53, 78, 79].

Dedicated, confidential post-incident clinician support was noted as a duty of employers, a necessary investment for normalising OD practices, and a crucial element for sustaining the wider trust and confidence of clinical teams and retaining staff, in two studies [31, 32]. However, the acceptability and availability of dedicated post-incident support systems to staff themselves remained unclear [32]. An EA identified from six studies [49, 50, 65, 69, 75, 78] posited that less formal workplace and peer support (if it happens without fear of blame or loss of reputation) is more relevant for OD improvement than formal training interventions, at least for to some health care professionals. Despite a vibrant social and organisational discourse on ‘open cultures’ and ‘fair cultures’ in healthcare, there was relatively limited discussion in the included documents of how these values and practices impinge on OD in maternity care [32].

#### Knowing that improvements are happening

##### Initial programme theory

When families and staff can see that aspects of a service are improving as a result of learning from the tragedy that has affected them, they are more likely to be able to deal with loss and trauma in the longer term and are less likely to feel alienated from the service.

Ten EAs, identified from 23 documents [6, 29–32, 34, 49–52, 59, 61, 63, 65, 72, 75–78, 80–82] identified a relationship between OD and post-incident learning, with outcomes described for families (*n* = 3); for staff (*n* = 4) and for services (*n* = 3). Many families anticipated that an incident review would both explain what happened in their case, and that this knowledge would be used to prevent the same thing from happening again in the future [61]. Assurance that a similar incident has been prevented in the future – and that their own experiences have contributed to this prevention- was found to help families to make sense of their loss [50, 52, 59, 81]. However, family expectations of improvements from learning were often not met [50, 52, 80], either because changes had not happened, were happening gradually, or were not communicated to the family [50, 65]. One UK study found that 83% of families felt that their incident investigation had made no positive difference to the service and 73% of families were unclear on what learning had happened [50].

Four EAs identified the importance of well-functioning, clinical governance systems to both ensure systems-level learning and to embed OD processes. In some cases, it was implied that this learning might include the incorporation of family oversight, perspectives, and experience. However, the significance of capacity in clinical or organisational teams to keep families updated on whether commitments to improvement were being met was also noted [30, 32, 34]. Organisational changes to facilitate the shift towards ongoing service improvement included strengthening assurance systems with regular reviews, implementing unit reporting for external benchmarking for ‘candour training,’ and increasing guideline compliance to promote learning and acting on lessons [31, 51, 75]. The clarification of service commissioners’ and Trust Board members’ responsibilities for meeting OD guidance or candour regulation, for enhancing family involvement in reviews and investigations, and for completing assurance of recommended action plans from these incidents was noted in a few studies [31, 32, 50]. However, the quality assurance frameworks supporting these systems could not sustain, and in some cases, undermined the practical ethics of openness and learning [32, 49–52, 78, 82]. For example, this ethos might guide Board or inspectors’ decisions to revise quality assurance measures, such as completing a review in the recommended timeframe or demonstrating that ongoing learning from incidents has been embedded in improvement outcomes, but this may take place unevenly and over longer periods [50].

Comprehensive reviews of whole care pathways, requiring multi-disciplinary and cross-service contribution, were noted as especially valuable for maximising possibilities for learning within and beyond maternity care. Particularly in situations where harm was less immediately obvious, collaborative learning networks beyond maternity care, such as networks that included primary care providers, enabled learning conversations to reduce misunderstanding and treatment delays for individual women and families. Such networks relied on material and social investment in cross-sector relationships. In particular, cross-sector working required the clarification of leadership responsibilities, reporting timelines, peer-review and ‘fresh-eyes’ contributions, and agreement on investigation methodologies, along with administrative co-ordination. Further service investment in review and investigation data with external quality improvement bodies and the dissemination of learning from these external bodies through services, units, and teams was expected to enhance learning for safety revisions beyond single organisations. The more complex task of translating these lessons into ongoing practice and systems revisions was rarely addressed in the literature. One study detailed the pivotal role of an influential professional and professional body in promoting members’ learning from their collaborative work with a woman who experienced unrecognised harm in maternity care [65].

## Discussion

This realist synthesis identifies five initial programme theories highlighting the factors that are required for successful OD in maternity settings from the perspective of three different interest groups (families; clinicians and services). Some of these factors have been previously identified, and are not unusual in studies of OD in general health care [10, 89–96] or more recent NHS England policy interventions [97]. Our realist synthesis adds to this, contributing detailed descriptions of the barriers and facilitators to this work across the entirety of the OD process for different stakeholders. From this perspective, we were able to explore how contexts, mechanisms, and outcomes interact within different aspects of OD, addressing our aim of identifying the critical aspects of OD and highlighting what works, for whom, how, and in what contexts. Additionally, our synthesis focused on a clinical and safety improvement arena where the effects of complex intervention and improvement efforts in post-incident communication play out in contexts where harm arising from health care is particularly profound and emotionally difficult, and sometimes uncertain. Here too, multiple improvement efforts can jostle for space.

In such circumstances, the critical factors underpinning the reasoning and resourcing of OD improvement can carry unintended implications for families, clinicians, and services. For example, the meaningful acknowledgement of harm to a family during an OD conversation with a clinician can later ring hollow when wider aspects of care or post-incident support or learning are felt to be lacking [98]. For those families who anticipate their personal experiences of an incident to affect change for others in the future, these legacies might be denied where family insights are not translated into knowledge for clinical or service improvement. Additionally, their incidents might not be prioritised for more efficient organisational learning. At the same time, sensitive invitations from a clinician or service for a family to discuss their experiences on their terms may disrupt the administrative pace and purpose of OD as an auditable output [10]. Further tensions emerge as families and clinicians rely on investigation findings to ‘make sense of things.’ Different frameworks require families and staff to negotiate and reconcile multiple sources of investigation activity and reporting. As diverse investigation approaches of the same incident draw different conclusions, the confidence of families and affected staff in service or wider investigating bodies can be compromised. At the same time, the ways that that potential discordance between investigator and family perspectives is approached by organisations indicates wider assumptions about the agency, expertise and entitlements of those most harmed by the incident.

Our focus on interventions intended to improve OD practice highlights a series of underlying assumptions about how educators and policymakers expect OD to happen, what underpins effective OD, and how improvements are fostered. We identified a wide range of interventions designed to strengthen OD in maternity settings. Overall, evidence regarding the effectiveness of interventions is weak, with limited possibilities for comparison. However, with notable exceptions [48, 65, 78], the included reports and papers included limited suggested changes for family involvement in understanding the incidents affecting them, despite over sixteen years of international improvement efforts. The included papers more often documented evidence of what improvements people want, rather than what improvements have happened and to what end.

One series of OD interventions were inserted within wider improvement programs or strategies intended to improve incident analysis or audit (see Table 2). Here, OD events were reduced to single components of tool kits or items for audit, with the question of how OD is conducted and experienced largely overlooked. In these interventions, OD was considered a predictable and reportable task rather than an ongoing relationship that might address the wider family need. The extent to which these approaches meet some families’ expectations of recognition and understanding of incidents of harm requires further exploration. Another series of OD improvement interventions focused more narrowly on clinician training and guidance for OD conversations. These interventions, conducted in educational rather than clinical settings, fail to consider the demands and unpredictability of unfolding OD conversations in pressurised, emotive, and distracting care environments [97]. Without recognition of the organisational and local workplace conditions in which OD conversations are initiated and unfold, responsibilities for OD improvements are assumed to reside with trained individuals. In contrast to more singular interventions, other studies described systems-wide interventions designed as forms of ‘culture change management’ for open OD improvement across local hospitals, units, or services. These studies anticipate that OD improvements will be slow-paced, uneven, and complex. Evaluation studies of this approach documented expected changes in staff attitudes towards openness and transparency, responsibility and risk, and family involvement more generally. In these approaches, OD improvements intersect with and inform a range of activities associated with clinical governance, maternity safety strategies, and improvements in families’ experience of maternity care. For example, in one large-scale, cross-organisational directive [78, 99], a ‘systems-based approach’ to OD improvement incorporated a range of technologies ranging from local policy development and implementation, training events, awareness-raising, to dedicated championship and leadership. These multiple initiatives were expected to stimulate gradual shifts in formal and informal workplace practices that included local translation and adjustment of protocols and guidance in relation to work settings and circumstances [100]. In these evaluations, the implementation of OD policy found that service managers formulated their local approaches in relation to strategic principles underpinned by a clear ethos and supported by coordinated guidance [48, 78]. The recent introduction of the Patient Safety Incident Response Framework in the English NHS [97] also incorporates an organisational strategic approach to the involvement of patients or families in investigations and governance of these processes [97]. Some of our included studies [6, 48, 76, 78, 83] considered a wider socio-political promise of OD as an ethical practice. As such, it encapsulates an ethos of care and communication with patients and families that includes consideration both of alternative forms of expertise and user entitlement [6, 96]. This ethos is challenged in situations of poor outcome [76, 101], when resources required to support harmed families and staff are eroded [95] and when expectations of learning for future improvement evaporate.

A significant feature of the documents included here is the limited consideration of family inclusion in areas of organisational practice considered to be critical for OD improvement, for example, organisational governance or quality and safety improvement work. There was limited consideration of the need for family representation on review/investigation and wider quality assurance committees. This way, an awareness of family priorities was promoted and sustained in organisations, and in ways that might carry ripple effects for service-user involvement in healthcare organisations more generally. However, this potential for service-user involvement in the strategic management of maternity services is not expected to be unproblematic. Our synthesised findings indicate that more radical changes in the assumptions of clinical professionals and organisational managers are required before family involvement in organisational planning and decision-making gains traction and becomes ‘taken-for-granted.’ Additionally, our analysis indicates that any legacy work ongoing with families in organisations, along with the sustained prioritisation of investment in requisite skills and resources for this work, is likely to be unevenly distributed across maternity provision. There is significantly limited evidence of revisions in clinical attitudes, knowledge and practice as an outcome of collaboration between harmed patients and provider organisations or professional bodies.

## Conclusion

This realist synthesis provides a fine-grained understanding of significant contexts, underlying factors, and effects of OD interventions and OD practices in international maternity settings between 2000–2021. The focus of the more recent documents in our synthesis was on OD interventions in English NHS settings, where public as well as family concerns have driven an accumulation of safety improvement initiatives and associated quality assurance measures. We unpack some of the challenges that can arise during the ongoing practice of incident OD, for families, staff, and service managers. These challenges revolve around the tensions that arise from policies that drive the standardisation of communication practices, the categorisation of harm, and organisational procedures, and the reflexive shaping of post-incident care with respect to family-centred needs and the particularities and uncertainties of clinical situations.

While our realist synthesis focused on improvement interventions directed towards individual professionals, teams or care provider organisations, we also indirectly identify the impact of wider social and professional attitudes and institutional structures on individual and organisational efforts to address ongoing shortfalls in post-incident communication with families. The ongoing marginalisation of families from commentary on the organisation and delivery of post-incident communication and care is particularly noteworthy. Additionally, we identify the difference between public or policy urgency for improvement and accountability, and organisational capacity for embedding OD practices and expertise within ongoing clinical care. However, we also question the expectation of policymakers that open disclosure, when effectively implemented, will satisfy a multitude of social and health policy interests ranging from patient justice to safety improvement and savings for services. A sharp-end perspective on incident OD, indicated from this synthesis, would question this promise of automatic mutual benefit. Instead, our synthesis suggests that the anticipated effects and valuations of incident OD are more fluid and differ in practice for different families, clinicians, and service representatives. People reflect and unite around their situated visions of what they should do and what they require when an incident of harm is considered [102]. A more detailed understanding of the various organisational and wider social spaces where these negotiations occur is required to better understand how underlying relationships and resources of acknowledgement, safety, advocacy, sense-making of an event, and learning are enacted in a rapidly changing and challenged maternity service.

This realist synthesis establishes the foundations of a primary research study that will explore, deepen or overturn the five initial programme theories by interview research with families, clinicians and health service managers, and by ethnographic investigation within NHS maternity services.

### Study strengths and limitations

Following realist principles [88, 103] a non-linear, iterative approach to data searching, along with the inclusion of heterogenous evidence sources, allowed this synthesis to develop and refine relevance during the data collection process. More traditional search strategies would have excluded many relevant sources that were not peer-reviewed. The strength of this analysis is that the identified underlying factors for OD improvement have been developed iteratively with input from expert stakeholders with differing perspectives, including health service policymakers, clinicians, third-sector leads, and families themselves. The co-investigator group, with OD expertise from a variety of backgrounds including social science, nursing, midwifery, medicine, and the third-sector, offered deeper insight into the subject. The initial programme theories have been constructed based on their pragmatic relevance in guiding future ethnographic research within maternity services [35]. At the same time, this approach included data that might have the same validity as data extracted for a traditional systematic review. We sought to ensure transparency of findings, however there are limitations to how far this is possible because of the iterative nature of realist data extraction and analysis. Most significantly, we note the tendency of documents and data to assume that families speak with one voice or that family differences are a marginal concern. While we have maintained a focus on international maternity settings, the most recent documents are from English health services, where there is a notable policy drive for maternity safety improvement where there is significant public pressure for improvements in openness with families and their inclusion in investigations [104, 105]. This may decrease the external validity of the results. These themes should be explored more widely in empirical research in both the English NHS and other health systems.

## Supplementary Information

**Additional file 1: Appendix 1.** Two-stage search strategy for realist synthesis.

**Additional file 2: Appendix 2.** Document appraisal for realist synthesis.

## Data Availability

The datasets generated and/or analysed during the current study are available from the corresponding author on reasonable request.

## Abbreviations

C-M–O: Context-Mechanism-Outcome
DoC: Duty of Candour
EA: Explanatory Account
NHS: National Health Service
OD: Open Disclosure
