# Supplementary material for: Strengthening open disclosure after incidents in maternity care: a realist synthesis of international research evidence
Source: BMC Health Serv Res. 2023 Mar 27;23:285. doi: 10.1186/s12913-023-09033-2 (PMC10041808; doi:10.1186/s12913-023-09033-2)
Supplement: Supplementary file 1 — Additional file 1: Appendix 1. Two-stage search strategy for realist synthesis. [file 12913_2023_9033_MOESM1_ESM.docx]

**APPENDIX 1: TWO-STAGE SEARCH STRATEGY FOR REALIST SYNTHESIS**

**STAGE 1**

1. **Literature Search in:**

MEDLINE; Embase; CINAHL; PsychINFO; Social Science Citiation Index; Wed of Science; HMIC; ERIC; ASSIA; the Cochrane Library (Register of Clinical Trials and Database of Systematic Reviews)

**Free Text and mapped terms and operators:**

“disclos*.mp” OR “candour” OR “communicat*.mp”

AND

“adverse event*.mp” OR “serious incident*.mp” OR “harm” OR “error.xxx”

AND

“strateg*mp” OR “interven*.mp” OR “training”

1. **Literature Searches for Grey Literature and Thesis Records in:**

OpenGrey; OpenSource; Google Scholar; Proquest and British Library EThOS

Each search undertaken separately for the following terms:

“open disclosure”; “disclosure”; “candour”; “duty of candour”; “communication of harm to patients”; “communication of incident to patients”; “communication adverse event to patients”

Stage 1 Exclusions: non-English language sources; documents published/available before 2000.

**STAGE 2:**

Free-text search of Stage 1 data-base (from above therefore includes Stage 1 exclusions) for following terms:

Matern*; obstetric*; midwife*; perinatal*; childbirth
