# Supplementary material for: Strengthening open disclosure after incidents in maternity care: a realist synthesis of international research evidence
Source: BMC Health Serv Res. 2023 Mar 27;23:285. doi: 10.1186/s12913-023-09033-2 (PMC10041808; doi:10.1186/s12913-023-09033-2)
Supplement: Supplementary file 2 — Additional file 2: Appendix 2. Document appraisal for realist synthesis. [file 12913_2023_9033_MOESM2_ESM.docx]

**APPENDIX 2: DOCUMENT APPRAISAL FOR REALIST SYNTHESIS**

Following RAMESES guidance, appraisal of the contribution of any section of data (within a document) is made following the two criteria of Relevance (whether the document or document sections can contribute to theory building or theory testing) and of Rigour (whether the method used to generate that particular piece of data is credible and trustworthy) (Wong et al 2013).

The Purpose of Ranking in relation to our DISCERN study objective:

to establish **initial hypotheses to focus the realist investigation of Open Disclosure** **improvements in NHS maternity services**; METHOD: Literature review and stakeholder consultation on OD interventions in maternity care (Published as an output); MONTHS:1-10

Relevance Ranking (adapted from CASP):

1. Includes all elements of one or more C, M, and Os (with evidenced outcome(s)).
2. Includes all elements of one or more C, M and Os (not evidenced outcomes(s)).
3. Includes some but not all elements of any C, M or Os.
4. Includes some evidence of M (but no C or O)
5. Includes none of above

Rigour Ranking (adapted from Booth et al 2021 p.165):

1. Results of the item (paper/report/commentary) is valid; reliable and generalisable
2. Results of the item (paper/report/commentary) is generalisable
3. Results of the item (paper/report/commentary) is valid and reliable
4. Results of the item (paper/report/commentary) is valid
5. Results of the item are none of the above

Definitions for rigour ranking used (adapted by MA to include grey literature):

1. Generalisability: empirical (extent to which evidence discussed can be used to infer characteristics about a wider population (Mason 2002); or theoretical (extent to which evidence discussed can be used to develop concepts, phenomena or theoretical propositions relevant to another setting or social group (Draper 2004)
2. Validity: accurate or true account of the evidence included (assessed by evidence from other commentators; or by different bodies of evidence used (Murphy 2003)
3. Reliability: if conclusions from evidence presented would be the same for different writers (from Hammersley 1994).

**REFERENCES:**

- Booth, A., Sutton, A., Clowes, M. and Martyn-St James, M., 2021. Systematic approaches to a successful literature review.
- Draper, A.K., 2004. The principles and application of qualitative research. *Proceedings of the nutrition society*, *63*(4), pp.641-646.
- Hammersley, M., 1994. Interpreting Qualitative Data: Methods for Analysing Talk, Text and Interaction.
- Mason, L., 2002. Developing epistemological thinking to foster conceptual change in different domains. In *Reconsidering conceptual change: Issues in theory and practice* (pp. 301-335). Springer, Dordrecht.
- Murphy, K.R. ed., 2003. Validity generalization: A critical review.
- Wong, G., Westhorp, G., Pawson, R. and Greenhalgh, T., 2013. Realist synthesis. *RAMESES training materials. London: The RAMESES Project*.
